# Tumor Microenvironment Characterization for Assessment of Recurrence and Survival Outcome in Gastric Cancer to Predict Chemotherapy and Immunotherapy Response

**DOI:** 10.3389/fimmu.2022.890922

**Published:** 2022-04-29

**Authors:** Yan Chen, Zepang Sun, Li Wan, Hongzhuan Chen, Tieju Xi, Yuming Jiang

**Affiliations:** ^1^Shatou Community Health Service Center, Shenzhen Hospital of Integrated Traditional Chinese and Western Medicine, The Second People’s Hospital of Bao’an Shenzhen (Group), Shenzhen Bao’an Shajing People’s Hospital, Guangzhou Medical University, Shenzhen, China; ^2^Department of General Surgery, Nanfang Hospital, Southern Medical University, Guangzhou, Guangdong, China; ^3^Department of Radiation Oncology, Stanford University School of Medicine, Stanford, CA, United States

**Keywords:** tumor microenvironment, gastric cancer, recurrence, prognosis, chemotherapy, immunotherapy, classifier (classification tool)

## Abstract

**Background:**

The tumor microenvironment (TME) is crucial for tumor recurrence, prognosis, and therapeutic responses. We comprehensively investigated the TME characterization associated with relapse and survival outcomes of gastric cancer (GC) to predict chemotherapy and immunotherapy response.

**Methods:**

A total of 2,456 GC patients with complete gene-expression data and clinical annotations from twelve cohorts were included. The TME characteristics were evaluated using three proposed computational algorithms. We then developed a TME-classifier, a TME-cluster, and a TME-based risk score for the assessment of tumor recurrence and prognosis in patients with GC to predict chemotherapy and immunotherapy response.

**Results:**

Patients with tumor recurrence presented with inactive immunogenicity, namely, high infiltration of tumor-associated stromal cells, low infiltration of tumor-associated immunoactivated lymphocytes, high stromal score, and low immune score. The TME-classifier of 4 subtypes with distinct clinicopathology, genomic, and molecular characteristics was significantly associated with tumor recurrence (P = 0.002), disease-free survival (DFS, P <0.001), and overall survival (OS, P <0.001) adjusted by confounding variables in 1,193 stage I–III GC patients who underwent potential radical surgery. The TME cluster and TME-based risk score can also predict DFS (P <0.001) and OS (P <0.001). More importantly, we found that patients in the TMEclassifier-A, TMEclassifier-C, and TMEclassifier-D groups benefited from adjuvant chemotherapy, and patients in the TMEclassifier-B group without chemotherapy benefit responded best to pembrolizumab treatment (PD-1 inhibitor), followed by patients in the TMEclassifier-A, while patients in the C and D groups of the TMEclassifier responded poorly to immunotherapy.

**Conclusion:**

We determined that TME characterization is significantly associated with tumor recurrence and prognosis. The TME-classifier we proposed can guide individualized chemotherapy and immunotherapy decision-making.

## Introduction

Gastric cancer (GC) is the fifth most common malignancy and the third leading cause of cancer-related deaths in the world (1). Despite significant advances in early diagnosis and treatment, the 5-year overall survival (OS) rate for patients with local GC remains around 40% in western countries (2, 3). Furthermore, most patients with GC die of tumor recurrence or metastasis (1–3). It has been reported that about 40% of GC patients relapse within 2 years after surgery, which has often been found in peritoneal, hematogenous, and nodal metastases (4, 5). Moreover, the median survival time from recurrence to death is approximately 4–6 months (5, 6). Additionally, with early diagnosis of GC, radical surgery is being performed more frequently, which has resulted in a rapid increase in relapse. Worse yet, there are no effective therapies for GC recurrence (1–7). Therefore, the prediction of GC recurrence in clinical practice seems to be of great importance.

The widespread application of genomic sequencing technology in tumor studies has provided us with possibilities for us to dissect the potential characteristics of the tumor microenvironment (TME) in GC (8–10). The TME, composed of the extracellular matrix, signaling molecules, and non-tumor cells, is heterogeneous. A growing body of evidence has suggested that TME is crucial in tumor progression and therapeutic responses (11–14). For instance, different compositions of tumor-associated cells infiltrating, namely, cytotoxic T cells, follicular helper T cells, natural killer cells, dendritic cells, tumor-associated macrophages, and cancer-associated fibroblasts, in TME are associated with diverse clinical outcomes, chemotherapy benefits, and immunotherapy responses (12–16). Thus, a more in-depth understanding of TME is indispensable and urgent. However, to date, the comprehensive landscape of TME characteristics in tumor recurrence has not been elucidated.

Evaluation of tumor recurrence in GC presents a major challenge. Traditional imaging modalities, such as X-ray, computed tomography (CT), Positron Emission Tomography-Computed Tomography (PET-CT), or ultrasonography, yield unsatisfactory predictions of GC recurrence (17). Additionally, these techniques could only detect metastases after their occurrence, thus delaying treatments. Using liquid biopsy, such as circulating tumor DNA, and radiomics, are the emerging means for detecting recurrence (18, 19). However, both are technically demanding and prone to interference currently. Thus, assessment of GC recurrence-associated TME characteristics and identification of patients at high risk of relapse after definitive therapy have great significance. Increased surveillance and early intervention may improve the quality of life and survival for these patients.

The heterogeneous nature of GC results in diverse clinicopathological and molecular features, which generates greater challenges to individualized diagnosis and treatment (20). Although chemotherapy and immunotherapy have improved survival in some GC patients, they are associated with unavoidable side effects (4, 21). However, only 9% absolute benefit is observed from adjuvant chemotherapy compared with the surgery-only group, and only 10–26% objective response rate is achieved in GC patients treated with immune checkpoint blockade (ICB), such as anti-programmed cell death protein 1 (PD-1) and anti-programmed death-ligand 1 (PD-L1) (4, 21–23). A considerable number of patients do not benefit from these treatments, suggesting that they could be spared from excessive intervention. Therefore, it is rewarding to identify patients with a response or resistance to specific treatments before initiation.

Recently, several molecular classification systems have been proposed for individualized diagnosis and treatment based on whole-genome and transcriptome data (8, 24–26). The Asian Cancer Research Group (ACRG) divided GC into 4 subtypes: microsatellite instability (MSI), mesenchymal transition (EMT), microsatellite stability/the tumor protein 53-active (MSS/TP53^+^), and microsatellite stability/the tumor protein 53-inactive (MSS/TP53^−^) (24). Alternatively, the Cancer Genome Atlas (TCGA) proposed four molecular subtypes: Epstein–Barr virus (EBV)-positive, microsatellite instability (MSI), genomically stable (GS), and chromosomal instability (CIN) (25). Subsequent studies defined other classification models based on tumor-associated infiltrating cells or mesenchymal–epithelial phenotypes (8, 24). However, the association between TME and GC recurrence has not been thoroughly explored in these studies.

The widely used algorithms, namely, CIBERSORT, MCPcounter, and ESTIMATE, have enabled us to explore the relationship between TME and tumor recurrence, survival, and therapeutic responses from bulk gene expression data recently (27–29). However, there are some differences in the cell compositions and data types calculated using different algorithms. Thus, a combination of multiple algorithms can complement each other and strengthen the conclusion, which may provide a better characterization of the TME.

In this work, we systematically evaluated the cellular component and prognostic landscape of the TME associated with GC relapse using three proposed computational algorithms (27–29). We next proposed a TME classifier composed of 4 subtypes associated with tumor recurrence and then validated its prognostic value for disease-free survival (DFS) and overall survival (OS) in multiple independent cohorts with 2,411 patients. Moreover, a novel classification system was observed with distinct clinicopathology, molecular, genomic, and epigenetic characteristics. Furthermore, we developed a TME-based risk score to predict DFS and OS, and confirmed that a TME-cluster of 3 phenotypes could predict DFS. Importantly, the TME classifier of the 4 subtypes we proposed could predict chemotherapy and immunotherapy responses.

## Materials and Methods

### Gastric Cancer Datasets and Clinical–Genomic Information

We conducted a systematic search for GC gene expression dataset, which were publicly accessible and had complete clinical annotations. Cases without survival information were excluded from further analysis. Totally, we achieved eleven cohorts of 2,411 patients with GC for this study: GSE62254/ACRG, GSE26253/SMC, GSE13861/YUHS, GSE26899/KUGH, GSE26901/KUCM, GSE15460/SGP, GSE28541/MDACC, GSE29272/TYB, GSE57308/CGH, GSE84437/KOREA, and TCGA-STAD. Raw data for the microarray datasets generated by Affymetrix or Illumina platform were screened from the Gene-Expression Omnibus (GEO; https://www.ncbi.nlm.nih.gov/geo/), and processed for background adjustment, quantile normalization, and final summarization by Perl software and limma packages. The corresponding clinical information was downloaded or manually registered from the item page in the GEO dataset website. For some series whose clinical data could not be obtained through the aforementioned methods, we retrieved the exact clinical information from the supplementary materials of relevant published papers (26, 30, 31). Level 4 gene-expression profile (FPKM normalized) and corresponding clinical data of The Cancer Genome Atlas (TCGA) were downloaded from the Genomic Data Commons (https://portal.gdc.cancer.gov/). Missing or updated clinical–genomic data was replenished from the UCSC Xena browser (GDC hub: https://gdc.xenahubs.net) and cBio Cancer Genomics Portal (cBioPortal: https://www.cbioportal.org/). DFS was defined as the time to recurrence at any site. OS was defined as the time to death from any cause. Among them, relapse information was recorded for six cohorts of ACRG, SMC, YUHS, KUGH, KUCM, and TCGA-STAD.

All patients except for the SGP cohort were reported with or without an operation note. Most patients were classified according to the American Joint Committee on Cancer (AJCC) 6th edition or 7th edition (32, 33). We redefined patients into the AJCC 8th edition based on the status of tumor-node-metastasis (TNM) available when necessary, with accuracy as the first criterion (34). Under these circumstances, we defined four meta-cohorts (A, B, C, and D) using cases with specific characteristics from the aforementioned eleven cohorts. The meta-cohort A (1,193 patients) included stage I–III patients with relapse records after potential radical surgery based on the AJCC 8th edition. The meta-cohort B (1,365 patients) included stage I–IV patients from six cohorts of ACRG, SMC, YUHS, KUGH, KUCM, and TCGA-STAD. The meta-cohort C (1,046 patients) included stage I–IV patients from five cohorts of SGP, MDACC, TYB, CGH, and Korea. The meta-cohort D (2,411 patients) consisted of meta-cohorts B and C. Adjuvant chemotherapy was mainly fluorouracil-based and was available in six cohorts: ACRG, SMC, YUHS, KUGH, KUCM, and MDACC.

### Gastric Cancer Dataset With PD-1 Inhibition Treatment

The original paired gene sequence and corresponding clinical information of the PRJEB25780 cohort (pembrolizumab treatment) were downloaded from the European Bioinformatics Institute (EMBL-EBI) database (https://www.ebi.ac.uk/). The adapter and low-quality sequences were removed from the raw data using Trim Galore software. The quality of the samples after filtration was checked and adjusted by the FastQC software. Clean reads were compared with the human genome (HG38 version) using HISAT2 software, and then a read count of gene expression was generated using FeatureCounts software. Finally, the gene expression profile was normalized by the limma package.

### Tumor Microenvironment Infiltrating Cells Dissecting

To quantify the composition of tumor-associated infiltrating cells in the GC samples, two proposed computational algorithms, CIBERSORT and Microenvironment Cell Populations-counter (MCPcounter), were conducted (27, 28). Based on the gene expression profiles, the CIBERSORT algorithm was employed to quantify the proportions of tumor-infiltrating immune cells using standard reference files at parameter settings of LM22 signature and 1,000 permutations (27). A total of 22 types of immune cells, namely, naive B and memory B cells, CD8^+^ T cells, naive CD4^+^ T cells, resting memory CD4^+^ T cells, activated memory CD4^+^ T cells, T follicular helper cells (Tfh), regulatory T cells (Tregs), resting natural killer (NK) cells, activated NK cells, M0 macrophages, M1 macrophages, M2 macrophages, resting dendritic cells (DC), activated DC, resting mast cells, activated mast cells, plasma cells, gamma delta T cells, monocytes, neutrophils, and eosinophils, were obtained through this algorithm (27). Additionally, the absolute abundance of ten kinds of immune-stromal associated cells, including two stromal cells (tumor-associated fibroblasts and endothelial cells) and eight immune cells (CD3 T cells, CD8 T cells, cytotoxic lymphocytes, B cell lineage, NK cells, monocytic lineage, myeloid dendritic cells, and neutrophils), was estimated by the MCPcounter algorithm (28).

### Discovery and Validation of the TME-Classifier

The tumor-associated immune and stromal scores representing TME characterization were calculated based on the normalized gene-expression matrix using the ESTIMATE algorithm for each GC sample (29). Subsequently, for each dataset, the expression of the immune-stromal score was transformed into a z-score. We then classified patients into a high-immune group and a low-immune group or a high-stroma group and a low-stroma group using a score of 0 as the cutoff, as described in our published paper (11). Furthermore, we developed a TME-classifier of 4 subtypes based on the aforementioned results: TMEclassifier-A (low-immune and low-stroma score), TMEclassifier-B (high-immune and low-stroma score), TMEclassifier-C (low-immune and high-stroma score), and TMEclassifier-D (high-immune and high-stroma score). Finally, the TME-classifier of 4 subtypes was validated in 12 independent cohorts and 4 meta-cohorts to assess GC recurrence, clinical–genomic characteristics, components of tumor-associated infiltrating cells, DFS, OS, chemotherapy, and immunotherapy responses.

### TME-Cluster and TME-Based Risk Score Developing

To further dissect the association between TME characteristics and GC recurrence, we employed an unsupervised consensus clustering algorithm on 22 tumor-associated infiltrating immune cells and 2 tumor-associated infiltrating stromal cells, whose values had been standardized by Z-score and defined the robust subgroup of patients (35). Next, given the diverse prognostic value of the immune and stromal scores described previously, we integrated them into a comprehensive TME-based risk score. The TME risk score was calculated using the following equation: TME risk score = tumor-associated stromal score − tumor-associated immune score. Both the TME-cluster and TME-based risk scores were used to evaluate DFS and OS. Similarly, the TME-cluster of 3 subtypes was used to predict chemotherapy and immunotherapy responses.

### Functional Enrichment Analysis

Gene annotation enrichment analysis was performed to gain an in-depth understanding of tumor microenvironment characteristics using the R package clusterProfiler (36). An adjusted P-value of <0.05 was identified as significant. Considering the quantity, quality, completeness, and representativeness of the datasets, gene set enrichment analyses (GSEA) were performed in the TCGA-STAD and KOREA cohorts.

### Prediction of Chemotherapy and Immunotherapy Response

We next evaluated the predictive performance of our TME-classifier and TME-cluster on chemotherapy and immunotherapy responses in a meta-cohort of 903 GC with chemotherapy information and the PRJEB25780 cohort with anti-PD-1 treatment. Specifically, Kaplan–Meier curves for overall survival were performed to decide which subgroup of TME-classifier and TME-cluster could benefit from chemotherapy. Moreover, the rate of immunotherapy response, including an objective response rate (ORR), was calculated to be observed in which subgroups of TME-classifier and TME-cluster could benefit from immunotherapy. Meanwhile, the area under the receiver operating characteristic curve (AUC) was used to assess the predictive power.

### Statistical Analysis

Continuous variables were compared among groups using the t-test, Mann–Whitney U test, or Kruskal–Wallis test. Enumeration data were compared among groups by the Chi-square or Fisher exact test. All statistical analyses were performed using R software (version 3.5.3), origin software (version 2019b) and SPSS statistical software (version 24.0). A two-sided of P <0.05 was considered statistically significant.

## Results

### Tumor Microenvironment Characterization Associated With Cancer Recurrence

A total of 2,456 patients from 12 cohorts were included in this study. The clinicopathological and treatment information is presented in **Table 1** and **Table S1**. After preprocessing, 1,193 stage I–III patients with relapse records after potential radical surgery were identified from 6 cohorts, which included ACRG (n = 257), SMC (n = 365), YUHS (n = 55), KUCM (n = 102), KUGH (n = 85), and TCGA-STAD (n = 329). These cohorts and an integrated meta-cohort were used to assess the association between the TME characteristics and tumor recurrence. On the whole, our data showed that patients without recurrence at the last follow-up had an active immune response and inactive immunosuppression (**Figures S1**, **S2**). Specifically, significantly higher infiltration of CD4 activated cells, NK activated cells, plasma cells, but significantly lower infiltration of M2 macrophages in patients without recurrence in cohorts of ACRG, TCGA-STAD, SMC, KUCM, and KUGH, separately (**Figure S3A**). Additionally, patients with recurrence exhibited a significantly higher abundance of tumor-associated stromal cells, including fibroblasts and endothelial cells, in all cohorts, especially ACRG, SMC, KUCM, and KUGH (**Figures S2**, **S3B**). Importantly, and believably, a higher stromal score and a lower immune score were found in patients with recurrence (**Figures S3C, D**).

**Table 1:** Clinicopathologic and treatment information of patients with gastric cancer in eleven public cohorts.

Variables	ACRG	SMC	YUHS	KUCM	KUGH	TCGA-STAD	SGP	MDACC	TYB	CGH	KOREA
**No. of patients**	300	432	59	109	93	372	248	40	126	199	433
**Median age (range)**	64 (24–86)	53 (23–74)	63 (32–83)	58 (28–74)	60 (36–83)	67 (35–90)	68 (23–92)	58 (33–78)	59 (23–71)	61 (34–81)	62 (27–86)
**Male (%)**	199 (66.3)	280 (64.8)	41 (69.5)	69 (63.3)	73 (78.5)	240 (64.5)	161 (64.9)	27 (67.5)	99 (78.6)	145 (72.9)	296 (68.4)
**Location (%)**											
Cardia	32 (10.7)	54 (12.5)	4 (6.8)	13 (11.9)	9 (9.7)	133 (35.8)	–	–	–	–	–
Body	107 (35.7)	139 (32.2)	28 (47.5)	36 (33.0)	29 (31.2)	89 (23.9)	–	–	–	–	–
Antrum	155 (51.7)	226 (52.3)	24 (40.7)	56 (51.4)	55 (59.1)	134 (36.0)	–	–	–	–	–
Whole	6 (2.0)	13 (3.0)	1 (1.7)	4 (3.7)	–	–	–	–	–	–	–
**Lauren type (%)**											
Intestinal	150 (50.0)	139 (32.2)	17 (28.8)	82 (75.2)	59 (63.4)	162 (43.5)	138 (55.6)	–	–	55 (27.6)	–
Diffuse	141 (47.0)	280 (64.8)	28 (47.5)	11 (10.1)	31 (33.3)	74 (19.9)	86 (34.7)	–	–	101 (50.8)	–
Mixed	9 (3.0)	13 (3.0)	12 (20.3)	5 (4.6)	2 (2.2)	–	22 (8.9)	–	–	43 (21.6)	–
**T stage (%)**											
T1	0	–	–	–	–	20 (5.4)	–	–	–	1 (0.5)	11 (2.5)
T2	188 (62.7)	–	–	–	–	80 (21.5)	–	–	–	25 (12.6)	38 (8.8)
T3	91 (30.3)	–	–	–	–	170 (45.7)	–	–	–	145 (72.9)	92 (21.2)
T4	21 (7.0)	–	–	–	–	98 (26.3)	–	–	–	28 (14.1)	292 (67.4)
**N stage (%)**											
N0	38 (12.7)	–	–	–	–	114 (30.6)	–	–	–	42 (21.1)	80 (18.5)
N1	131 (43.7)	–	–	–	–	101 (27.2)	–	–	–	72 (36.2)	188 (43.4)
N2	80 (26.7)	–	–	–	–	75 (20.2)	–	–	–	66 (33.2)	132 (30.5)
N3	51 (17.0)	–	–	–	–	75 (20.2)	–	–	–	19 (9.5)	33 (7.6)
**M stage (%)**											
M0	273 (91.0)	–	55 (93.2)	102 (93.6)	85 (91.4)	347 (93.3)	–	–	–	175 (87.9)	–
M1	27 (9.0)	–	4 (6.8)	7 (6.4)	7 (7.5)	23 (6.2)	–	–	–	24 (12.1)	–
**TNM stage (%)**											
I	30 (10.0)	68 (15.7)	12 (20.3)	40 (36.7)	11 (11.8)	52 (14.0)	42 (16.9)	1 (2.5)	5 (4.0)	9 (4.5)	–
II	97 (32.3)	167 (38.7)	11 (18.6)	18 (16.5)	18 (19.4)	112 (30.0)	42 (16.9)	6 (15.0)	5 (4.0)	31 (15.6)	–
III	96 (32.0)	130 (30.1)	24 (40.7)	36 (33.0)	27 (29.0)	155 (41.7)	91 (36.7)	12 (30.0)	108 (85.7)	104 (52.3)	–
IV	77 (25.7)	67 (15.5)	12 (20.3)	15 (13.8)	36 (38.7)	39 (10.5)	73 (29.4)	21 (52.5)	8 (6.3)	55 (27.6)	–
**Potential radical surgery**	Reported	Reported	Reported	Reported	Reported	Reported	Not reported	Reported	Reported	Reported	Reported
**Chemotherapy (%)**	144 (48.0)	432 (100)	45 (76.3)	39 (35.8)	67 (72.0)	–	–	40 (100)	–	–	–
**Recurrence (%)**	125 (41.7)	177 (41.0)	26 (44.1)	60 (55.0)	32 (34.4)	123 (33.1)	–	–	–	–	–
**Median follow-up, mo.**	80	84	91	94	49	22	75	85	70	51	116

A dash indicates that data are not available. The version of TNM staging system for ACRG, SMC, YUHS, KUCM, KUGH, TCGA, SGP, MDACC, TYB, CGH, and KOREA was the American Joint Committee on Cancer (AJCC) 6th or 7th edition.

### TME-Classifier Can Predict Disease-Free Survival and Overall Survival Independent of the TNM Staging

**Figure 1:**
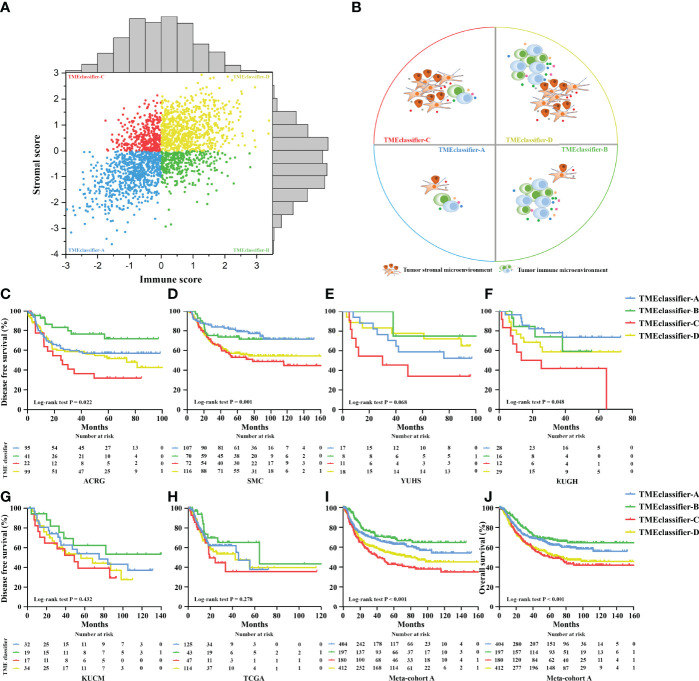
Patients were divided into 4 subtypes of TME-classifier, based on the immune score and stromal score **(A, B)**. Kaplan–Meier curves for disease-free survival of patients with stage I–III gastric cancer after potential radical surgery based on TME-classifier in the ACRG **(C)**, SMC **(D)**, YUHS **(E)**, KUGH **(F)**, KUCM **(G)**, and TCGA **(H)** cohorts. Kaplan–Meier curves for disease-free survival **(I)**, and overall survival **(J)** of 1,193 patients with stage I–III gastric cancer after potential radical surgery based on TME-classifier in the meta-cohort A.

**Figure 2:**
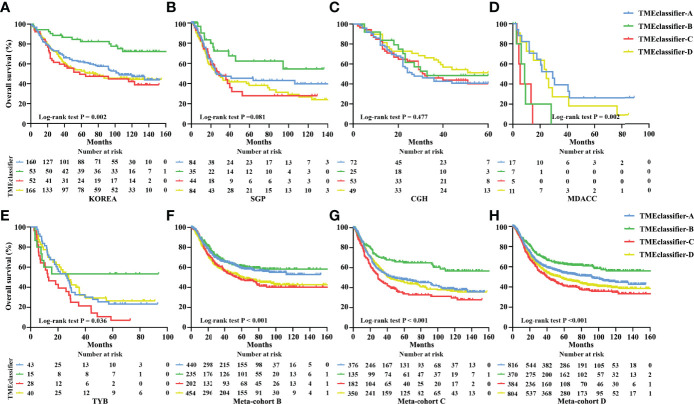
Kaplan–Meier curves for overall survival of patients with stage I–IV gastric cancer based on TME-classifier in the KOREA **(A)**, SGP **(B)**, CGH **(C)**, MDACC **(D)**, and TYB **(E)** cohorts. Kaplan–Meier curves for overall survival **(F)** of 1,365 patients with stage I–IV gastric cancer based on TME-classifier in the meta-cohort B Kaplan–Meier curves for overall survival **(G)** of 1,046 patients with stage I–IV gastric cancer based on TME-classifier in the meta-cohort C Kaplan–Meier curves for overall survival **(H)** of 2,411 patients with stage I–IV gastric cancer based on TME-classifier in the meta-cohort D.

Next, we developed the TME-classifier, which divided patients into 4 subtypes based on the low-high immune and low-high stromal scores (**Figures 1A, B**). **Table S2** contains the exact information of the TME-classifier for 11 cohorts. Most importantly of all, we demonstrated that the TMEclassifier was a robust prognostic biomarker for DFS and OS in multiple cohorts of ACRG, SMC, YUHS, KUGH, KUCM, TCGA, KOREA, SGP, CGH, MDACC, TYB, meta-cohort A, meta-cohort B, meta-cohort C, and meta-cohort D (**Figures 1C–J** and **2A–H**). Although statistical significance was not found in some cohorts due to a small sample or short follow-up period, there was a similar tendency in prognosis: the TMEclassifier-B showed the best prognosis, followed by the TMEclassifier-A, TMEclassifier-D, and then TMEclassifier-C. **Tables S3**–**13** contain a summary of these findings. More importantly, when integrated into a meta-cohort A, a meta-cohort B, a meta-cohort C, and a meta-cohort D, a more robust outcome for TMEclassifier to predict DFS (P <0.001) and OS (P <0.001) in univariate and multivariate Cox analyses was confirmed (**Table S14** and **Table 2**), as demonstrated by the Kaplan–Meier curves (**Figures 1**, **2**).

**Table 2:** Multivariable cox regression analyses for disease-free survival and overall survival in gastric cancer meta-cohort.

Variables	Disease-free survival		Overall survival	
	HR (95%CI)	P	HR (95%CI)	P
**Age**	1.010 (1.003–1.020)	0.047	1.008 (1.000–1.017)	0.062
**Location**				
Cardia	–	–	1	Reference
Body	–	–	1.043 (0.759–1.434)	0.793
Antrum	–	–	0.882 (0.645–1.205)	0.430
Whole	–	–	1.208 (0.659–2.215)	0.540
**TNM stage**				
I	1	Reference	1	Reference
II	3.100 (2.038-4.715)	<0.001	3.268 (2.153–4.960)	<0.001
III	5.687 (3.692–8.760)	<0.001	5.867 (3.815–9.022)	<0.001
IV	–	–	10.769 (6.848–16.935)	<0.001
**Chemotherapy**				
No	1	Reference	1	Reference
Yes	0.494 (0.386–0.633)	<0.001	0.446 (0.357–0.556)	<0.001
**TMEclassifier**				
A	1	Reference	1	Reference
B	0.795 (0.554–1.142)	0.214	0.808 (0.596–1.095)	0.169
C	1.697 (1.239–2.323)	0.001	1.463 (1.097–1.953)	0.010
D	1.392 (1.063–1.824)	0.016	1.300 (1.014–1.665)	0.038

CI, confidence interval; HR, hazard ratio. Patients for disease-free survival assessment had undergone potential radical surgery.

### Tumor Microenvironment Landscape of the TME-Classifier

**Figure 3:**
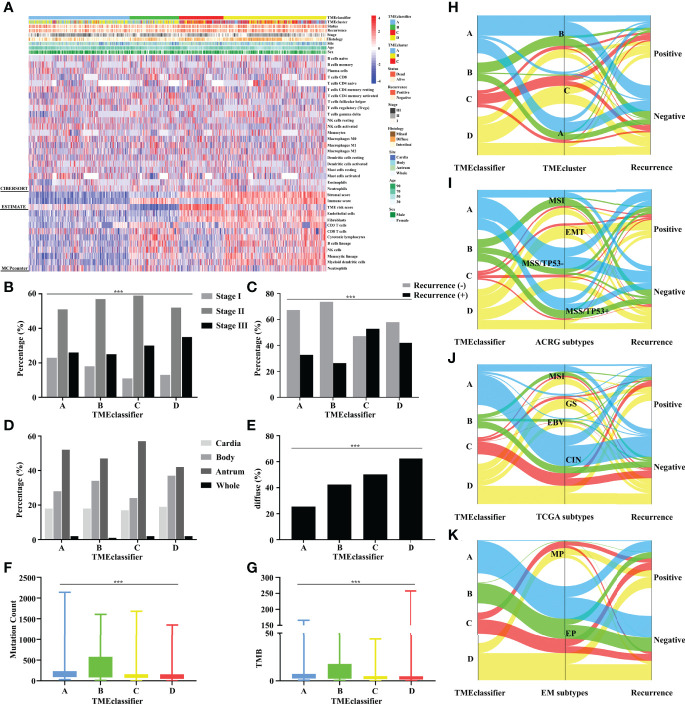
Unsupervised clustering of TME cells for 1,193 patients. TME-classifier, TME cluster, survival status, cancer recurrence, stage, histologic subtype, tumor site, age, and sex are shown as annotations **(A)**. TME-classifier differences in the TNM staging **(B)**, cancer recurrence **(C)**, tumor site **(D)**, and histologic subtype **(E)**. Mutation count **(F)** and TMB score **(G)** stratified by TME-classifier. Alluvial diagram of TME-classifier in groups with different TME clusters **(H)**, ACRG subtypes **(I)**, TCGA subtypes **(J)**, and EM subtypes **(K)**. ***P<0.001.

Based on the above phenomenon, we confirmed that TME characterization was closely associated with cancer recurrence and survival outcome. We then screened and analyzed the tumor-associated infiltrating immune-stromal cell landscape of the TME-classifier in 1,193 stage I–III patients with relapse information. Generally, compared with patients in the TMEclassifier-C + D group, we found that patients in the TMEclassifier-B group were characterized by a higher abundance of immunoactivating cells and a lower abundance of immunosuppressive cells. For example, a high abundance of CD8^+^ T cells, T follicular helper cells, memory CD4 T-activated cells, M1 macrophages, cytotoxic lymphocytes, and NK cells, while a low abundance of M2 macrophages, regulatory T cells, and mast cells were observed in patients of the TMEclassifier-B group (**Figure 3A** and **Figure S4**). Additionally, compared with patients in the TMEclassifier-B + C + D group, we found that patients in the TMEclassifier-A group presented the lowest abundance of immune-stromal cells, especially calculated by the MCPcounter algorithm, and the lowest immune-stromal score calculated by the ESTIMATE algorithm, which may be associated with immune deficiency (**Figure 3A** and **Figure S4**). Furthermore, compared with the TMEclassifier-A + B group, patients in the TMEclassifier-C + D group showed more characteristics of immunosuppression (high abundance of fibroblasts and endothelial cells, and high stromal score), especially in the TMEclassifier-C group (**Figure 3A** and **Figure S4**). Moreover, the findings suggested a complete immunosuppression phenomenon in the TMEclassifier-C group, while there was immunosuppression and immune-activation phenomenon (high immune score and limited immunoactivating cells) in the TMEclassifier-D group (**Figure 3A** and **Figure S4**). These results may further explain the different survival outcomes in the 4 subtypes of the TME-classifier.

### Assessing the Clinical, Genomic, and Molecular Characteristics Associated With the TME-Classifier

A higher proportion of earlier-stage GC patients was observed in the TMEclassifier-A + B group (**Figure 3B**). Moreover, the recurrence rate in the TMEclassifier-C + D group was significantly higher than that in the TMEclassifier-A + B group (**Figure 3C**). In particular, patients in the TMEclassifier-B group had the lowest recurrence rate. Similarly, we found that GC patients with cancer recurrence in 6 cohorts of ACRG, SMC, YUHS, KUCM, KUGH, and TCGA-STAD, had more patients in the TMEclassifier-C and TMEclassifier-D groups, but had fewer patients in the TMEclassifier-A and TMEclassifier-B groups (**Figure S5A**). Furthermore, when integrated into meta-cohort A, the number and percentage of TMEclassifier-A and TMEclassifier-B samples were still larger in patients without tumor recurrence (**Figures S5B, C**). In multivariable logistic regression analyses (**Table S15**), the TMEclassifier remained a significant predictor (P = 0.002) of GC recurrence after adjusting for clinicopathological factors. Likewise, the tumor location distribution among the 4 subtypes of TME-classifier was consistent (**Figure 3D**). Additionally, patients in the TMEclassifier-C + D group had a higher proportion of diffuse type GC (**Figure 3E**). Interestingly, the mutational load (mutation count and TMB) in the TMEclassifier-A + B group, especially group B, was significantly higher than that in the TMEclassifier-C + D group, showing an immunogenicity difference (**Figures 3F, G**). These statistical comparisons are presented in **Table S16**.

We also performed a comparison between our classification system and several existing molecular subtypes. Our findings showed that TMEcluster-A and -B groups had a higher degree of overlap with TMEclassifier-A and B groups, while almost all TMEcluster-C patients were observed in TMEclassifier-C and D groups (**Figure 3H** and **Figure S6A**). The EMT subtype of the ACRG classification with the worst prognosis had not been detected in patients of the TMEclassifier-A + B group, and patients in the TMEclassifier-B group presented the largest proportion of MSI and MSS/TP53^+^ subtype, followed by TMEclassifier-A (**Figure 3I** and **Figure S6B**). Additionally, we found significantly more patients with the EBV or MSI subtype of the TCGA classification in the TMEclassifier-B group, and a higher percentage of GS subtype was found in the TMEclassifier-C + D group (**Figure 3J** and **Figure S6C**). Meanwhile, there was almost no detection of any MP subtype in the TMEclassifier-A + B group, and the lowest proportion of EP cases was found in the TMEclassifier-C group (**Figure 3K** and **Figure S6D**). These results were highly consistent and significant, which confirmed the credibility and accuracy of our discovery.

### TME Cluster and TME-Based Risk Score Are Markers for Prognosis

**Figure 4:**
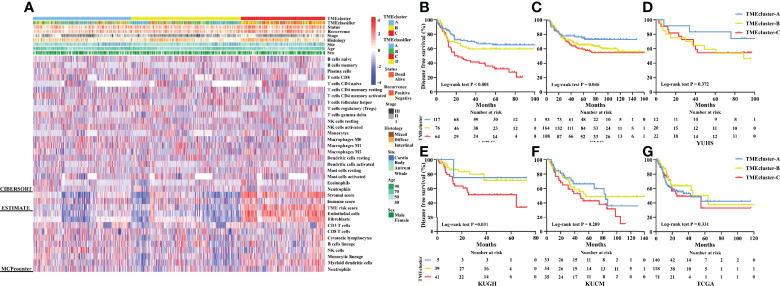
Unsupervised clustering of TME cells for 1,193 patients. TME cluster, TME-classifier, survival status, cancer recurrence, stage, histologic subtype, tumor site, age, and sex are shown as annotations **(A)**. Kaplan–Meier curves for disease-free survival of patients with stage I–IV gastric cancer based on TME-classifier in the ACRG **(B)**, SMC **(C)**, YUHS **(D)**, KUGH **(E)**, KUCM **(F)**, and TCGA **(G)** cohorts.

**Figure 5:**
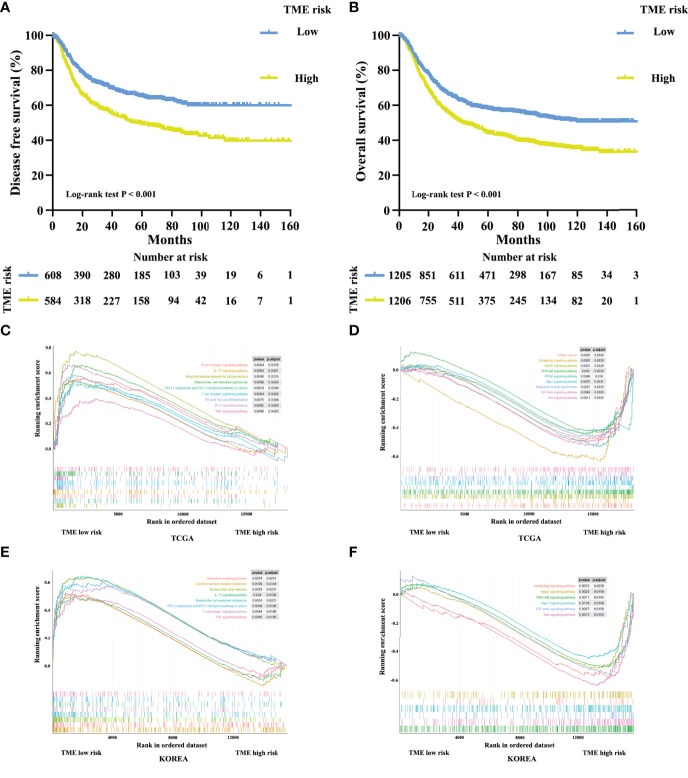
Kaplan–Meier curves for disease-free survival **(A)** of 1,193 patients with stage I–III gastric cancer after potential radical surgery based on TME risk score. Kaplan–Meier curves for overall survival **(B)** of 2,411 patients with stage I–IV gastric cancer based on TME risk score. Enrichment plots for upregulation **(C, E)** and downregulation **(D, F)** pathways of TME low risk group in the TCGA and KOREA cohorts.

Furthermore, we developed a TME cluster and a TME-based risk score as per the aforementioned description. The optimal number of clusters was found to be three, with maximal consensus within clusters and minimal ambiguity among clusters. When compared with the TMEcluster-C group, the TMEcluster-A group presented a higher abundance of immunoactivating cells and a lower abundance of immunosuppressive cells (immune-inflamed phenotype), while the TMEcluster-C group presented the highest abundance of stromal cells and the highest stromal score, which may be associated with an immune excluded phenotype. Moreover, the infiltration of both immune and stromal cells in the TMEcluster-B group was weak (immune-desert phenotype) (**Figure 4A** and **Figure S7**). We observed that the TME cluster can predict both DFS and OS in multiple cohorts and the meta-cohort as a supplement to the published results (**Figures 4B–G** and **Figure S8**). Additionally, patients were divided into low-risk and high-risk TME scores based on the median value. We found that patients with a high TME risk score had a poor prognosis, as shown by DFS and OS (**Figures 5A, B**).

### Underlying Mechanism of Tumor Microenvironment Characterization

A comprehensive GSEA analysis was performed between the low and high TME risk scores to identify the underlying mechanisms. We observed significant differences in inflammation-related pathways, chemokine pathways, tumor progression-associated pathways, and metabolic pathways between the low and high TME risk score groups. Patients in the low TME risk score group showed a preferable immunogenicity and tumor suppressive response, which was consistent with a better survival outcome. These results were supported by data from the TCGA and KOREA cohorts (**Figures 5C–F**).

### TME-Classifier Can Predict Chemotherapy and Immunotherapy Responses

**Figure 6:**
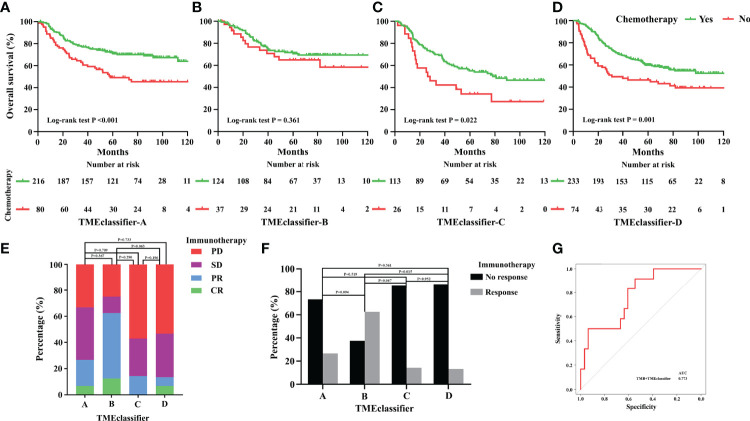
Predictive relevance of the TME-classifier for the benefit of chemotherapy in stage I–III gastric cancer. Patients of TME-classifier **(A, C, D)** derived a significant survival benefit from adjuvant chemotherapy **(A, C, D)**. However, patients of TME-classifier B did not benefit from adjuvant chemotherapy **(B)**. Patients in TME-classifier B group respond best to pembrolizumab treatment (PD-1 inhibitor), followed by TMEclassifier-A, TME-classifier C, and TME-classifier D groups **(E, F)**. TMEclassifier integrated with TMB score predict immunotherapy response well **(G)**.

Considering the important role of chemotherapeutic agents and immune checkpoint inhibitors in clinical application, we next explored patients whose subtype could benefit from chemotherapy and immunotherapy based on our TME-classifier and TME-cluster. Adjuvant chemotherapy resulted in a significant survival benefit for patients in the TMEclassifier-A, TMEclassifier-C, and TMEclassifier-D groups (**Figures 6A, C, D**), but not for patients in the TMEclassifier-B group (**Figure 6B**). Furthermore, forty-five GC patients with complete transcriptome matrix and clinical information were used to evaluate the TMEclassifier characterization for further analysis (**Table S1**). Interestingly, we found that patients in the TMEclassifier-B group (ORR: 62.5%) without chemotherapy benefit responded best to pembrolizumab treatment (PD-1 inhibitor), followed by the TMEclassifier-A group (ORR: 26.7%), while patients in the TMEclassifier-C (ORR: 14.3%) and TMEclassifier-D (ORR: 13.3%) groups responded poorly to immunotherapy (**Figures 6E, F**) (P <0.05). We further observed that our TMEclassifier integrated with the TMB score, with an AUC of 0.773, could predict immunotherapy response well (**Figure 6G**). Additionally, patients in the TMEcluster-A group responded well to chemotherapy and immunotherapy, while patients in the TMEcluster-C group responded to chemotherapy, and patients in the TMEcluster-B group responded poorly to both treatments (**Figure S9**).

## Discussion

In this study, we drew the TME landscape of GC recurrence and confirmed that TME characterization was significantly associated with tumor recurrence. Inactive immune responses and active stromal responses were observed in patients with GC recurrence. Subsequently, we proposed a TMEclassifier of 4 subtypes with distinct clinicopathology, epigenetic and molecular characteristics from multiple cohorts of 2,411 patients with GC. The TMEclassifier yielded results that were comparable with the existing molecular classification systems. Importantly, the TMEclassifier remained a robust prognostic biomarker of DFS and OS when adjusted for other clinical factors. We also confirmed that a TME cluster with 3 subtypes can predict DFS as a supplement to the published study. Furthermore, we developed a TME-based risk score and observed that patients with a high TME risk score had a poor prognosis as shown by DFS and OS. More importantly, we found that patients in the TMEclassifier-A, TMEclassifier-C, and TMEclassifier-D groups benefited from adjuvant chemotherapy, and patients in the TMEclassifier-B group without chemotherapy benefit responded best to pembrolizumab treatment (PD-1 inhibitor), followed by the TMEclassifier-A, while patients in the C and D groups of the TMEclassifier responded poorly to immunotherapy.

We observed that the immune-stromal score on the borderline between the subgroups of the TME-classifier was approximate, which made it difficult to classify patients near the cut-off value, a phenomenon common in any clinical marker. To solve this challenge, future studies should integrate multimodal data, such as radiomics. Moreover, a single algorithm, such as CIBERSORT, may have limitations in distinguishing similar cell types. However, our results, which were based on three algorithms, were similar or almost identical and were validated in 13 cohorts of 2,456 patients with GC, which indicated that our conclusion was relatively correct and well-supported.

Recently, an increasing number of studies have indicated that TME is important in tumor progression and therapeutic responses (11–14, 37–39). Furthermore, our previous works have shown that immune signature is an independent predictor for survival and chemotherapeutic benefits in GC (11, 12, 37, 39). Thompson et al. proposed that increasing CD8 cell infiltration was correlated with impaired PFS and OS in GC (40). On the other hand, Grunberg et al. observed that cancer-associated fibroblasts promoted gastric cancer progression (14). Additionally, Sakamoto et al. indicated that tumor-associated macrophages (M2) promoted peritoneal dissemination of GC (13). Compared with previous studies, ours had several following strengths. Firstly, considering a paucity of study examining the tumor recurrence of GC associated with TME or describing one immune-stromal cell simply (13, 14), this study drew the cellular component and prognostic landscape of the TME associated with GC relapse and survival outcome systematically. Secondly, this study included the largest sample, with 2,456 patients from multiple cohorts as validation to date. Thirdly, unlike previous studies, our study combined three independent computational algorithms to confirm that TME characterization was closely related to cancer recurrence.

Cancer recurrence is a fatal complication that compromises the survival and quality of life of patients with GC after comprehensive therapy (41). In a retrospective review of 1,172 GC patients who underwent radical surgery, 42% of patients developed recurrence, and 79% relapsed within two years (5). The median time to death from the time of recurrence was 6 months (5, 41). Currently, the prediction and diagnosis of GC recurrence mainly depends on TNM staging, clinical signs, medical imaging, or even reoperation during follow-up, which may result in delayed diagnosis and treatment (5, 6, 41–43). Our work adds to a growing body of evidence supporting the crucial role of the TME in cancer recurrence. Based on the novel classification model, patients in the TMEclassifier-C and D groups with a high risk of tumor recurrence deserve intensified therapeutic regimens and active surveillance to prevent cancer relapse and improve survival outcomes.

Although chemotherapy and immunotherapy are widely used in clinical practice currently, not all patients with GC can benefit from these treatments (4, 21–23). Disparate clinical outcomes have been observed among patients of the same TNM stage who received similar treatments (44, 45), suggesting that such patients should be given individualized interventions. In this study, we observed that patients in the TMEclassifier-B group with the largest proportion of MSI could not benefit from chemotherapy but had the best response to anti-PD1 treatment. Similarly, several international trials, namely, the MAGIC trial (International Standard Randomized Controlled Trial Number [ISRCTN] 93793971) and the CLASSIC trial (ClincalTrials.gov identifier NCT00411229), reported that patients having tumors with high MSI did not benefit from perioperative or adjuvant chemotherapy (46–48). Moreover, patients with tumors with high MSI, EBV, and mutation burden were more likely to obtain durable responses to immunotherapy (49–51). Our results agree well with these reports. However, a considerable number of patients who were chemotherapy-resistant and showed a response to immunotherapy in the TMEclassifier-B group had MSS or GS tumors, which may be a challenge to the existing hypothesis and needs to be investigated further. Lastly, our results suggested that patients in the TMEclassifier-A, C, and D groups were sensitive to chemotherapy and insensitive to immunotherapy. This indicated that our novel classification system could identify patients who had no therapeutic benefits to avoid the side-effects of adjuvant treatments, and conversely, other patients would receive aggressive regimens and frequent surveillance to prevent cancer recurrences and improve survival outcomes. We also observed that the TME-cluster could predict the response to chemotherapy and immunotherapy. However, these findings were based on a small sample size and a long treatment duration, which may have limited the quality of the observation. Future research is needed to confirm these discoveries.

Despite these findings, this study still has several limitations. Firstly, the primary point is its retrospective nature. Secondly, although multiple cohorts of 2,411 patients are included in this study, validation from our center is lacking and patients are waiting for it. Thirdly, because the immune-stromal data were generated from gene expression profiles, further in-depth data from immunohistochemistry, cell, or animal experiments are required to validate the present findings. Fourthly, patients on the borderline between the two groups are classified into a particularly distinct group, which should be verified with multimodal data in the future. Fifthly, the sample of patients who underwent chemotherapy and immunotherapy is small, and thus a large validation cohort is needed.

In conclusion, we drew a TME landscape on GC recurrence and confirmed that TME characterization was significantly associated with tumor recurrence. We then proposed a TMEclassifier of 4 subtypes with distinct clinicopathology, genomic and molecular characteristics from multiple cohorts of 2,411 patients with GC. Importantly, the TMEclassifier remained a robust prognostic biomarker of DFS and OS when adjusted for clinical factors. We also confirmed that a TME cluster with 3 subtypes and a TME-based risk score can predict DFS and OS. More importantly, we found that patients in 4 subtypes of TMEclassifier had different responses to chemotherapy and immunotherapy.

## Data Availability Statement

The datasets presented in this study can be found in online repositories. The names of the repository/repositories and accession number(s) can be found in the article/**Supplementary Material**.

## Ethics Statement

TCGA, GEO, and EMBL-EBI belong to public databases. The patients involved in the databases have obtained ethical approval. The studies were reviewed and approved by the Ethics Committee of Shenzhen Hospital of Integrated Chinese and Western Medicine and Nanfang Hospital of Southern Medical University. All procedures involving human participants were in accordance with the Declaration of Helsinki.

## Author Contributions

All authors listed had made substantial contribution to this work. YJ and YC conceived and designed the study. ZS, LW, HC, and TX collected and collated the data. YC and ZS analyzed the data and wrote the manuscript together. All authors listed have made a substantial, direct, and intellectual contribution to the work and approved it for publication.

## Funding

This work was supported by grants from the Natural Science Foundation of Guangdong Province (2019A1515011445), the National Natural Science Foundation of China (82102156) and the Sanming Project of Medicine in Shenzhen (No. SZZYSM202106009).

## Conflict of Interest

The authors declare that the research was conducted in the absence of any commercial or financial relationships that could be construed as a potential conflict of interest.

## Publisher’s Note

All claims expressed in this article are solely those of the authors and do not necessarily represent those of their affiliated organizations, or those of the publisher, the editors and the reviewers. Any product that may be evaluated in this article, or claim that may be made by its manufacturer, is not guaranteed or endorsed by the publisher.
