# Supplementary material for: Tumor Microenvironment Characterization for Assessment of Recurrence and Survival Outcome in Gastric Cancer to Predict Chemotherapy and Immunotherapy Response
Source: Front Immunol. 2022 Apr 29;13:890922. doi: 10.3389/fimmu.2022.890922 (PMC9101297; doi:10.3389/fimmu.2022.890922)
Supplement: Supplementary file 1 [file DataSheet_1.docx]

Supplementary Material

Tumor microenvironment characterization for assessment of recurrence and survival outcome in gastric cancer to predict chemotherapy and immunotherapy response

1. **Supplementary Methods**
2. **Supplementary Tables and Figures**
3. **Supplementary Reference**

**I Supplementary Methods**

1. CIBERSORT algorithm: CIBERSORT is a deconvolution algorithm [1] using a set of reference gene-expression values (a signature with 547 genes) considered a minimal representation for each cell type, and based on these values, infers cell type proportions in data from bulk tumor samples with mixed cell types using support vector regression. The CIBERSORT algorithm was employed to quantify the proportions of 22 Tumor-infiltrating immune cells using the LM22 signature and 1,000 permutations.

2. MCP-counter algorithm: Microenvironment Cell Populations-counter (MCP-counter) method [2], which allows the robust quantification of the absolute abundance of eight immune and two stromal cell populations in heterogeneous tissues from transcriptomic data.

3. ESTIMATE algorithm: ESTIMATE (Estimation of Stromal and Immune cells in MAlignant Tumour tissues using Expression data) algorithm [3] uses gene expression data to output the estimated levels of infiltrating stromal and immune cells and estimated tumour purity.  Stromal- and immune score are estimate based on 282 stromal-immune related genes.

4. TME-cluster developing: Unsupervised consensus clustering [4] is used to define the robust subgroup of patients. Specifically, the K-Means clustering algorithm with the Euclidean distance metric and performed 10,000 bootstraps, with 80% resampling of the immune-stromal cells.

**II. Supplementary Tables and Figures**

| Table S1. Clinicopathological characteristics of patients with gastric cancer in PRJEB25780 cohort. | | |
| --- | --- | --- |
| **Variables** | **N** | **%** |
| **Se** |  |  |
| Female | 13 | 28.9 |
| Male | 32 | 71.1 |
| **Recist** |  |  |
| CR | 3 | 6.7 |
| PR | 9 | 20.0 |
| SD | 16 | 37.8 |
| PD | 16 | 35.6 |
| **TMEclassifer** |  |  |
| A | 15 | 33.3 |
| B | 8 | 17.8 |
| C | 7 | 15.6 |
| D | 15 | 33.3 |

| Table S2. TMEclassifer information of patients with gastric cancer in all public cohorts. | | | | | | | | | | | |
| --- | --- | --- | --- | --- | --- | --- | --- | --- | --- | --- | --- |
| **Variables** | **ACRG** | **SMC** | **YUHS** | **KUCM** | **KUGH** | **TCGA** | **SGP** | **MDACC** | **TYB** | **CGH** | **KOREA** |
| **No. of patients** | 300 | 432 | 59 | 109 | 93 | 372 | 248 | 40 | 126 | 199 | 433 |
| **TMEclassifer (%)** |  |  |  |  |  |  |  |  |  |  |  |
| A | 109 (36.3) | 124 (28.7) | 17 (28.8) | 35 (32.1) | 29 (31.2) | 140 (37.6) | 84 (33.9) | 17 (42.5) | 43 (34.1) | 72 (36.2) | 161 (37.2) |
| B | 70 (23.3) | 82 (19.0) | 8 (13.6) | 20 (18.3) | 17 (18.3) | 47 (12.6) | 36 (14.5) | 7 (17.5) | 15 (11.9) | 25 (12.6) | 53 (12.2) |
| C | 22 (7.3) | 87 (20.1) | 13 (22.0) | 18 (16.5) | 13 (14.0) | 52 (14.0) | 44 (17.7) | 5 (12.5) | 28 (22.2) | 53 (26.6) | 52 (12.0) |
| D | 99 (33.0) | 139 (32.2) | 21 (35.6) | 36 (33.0) | 34 (36.6) | 133 (35.8) | 84 (33.9) | 11 (27.5) | 40 (31.7) | 49 (24.6) | 167 (38.6) |

| Table S3. Univariate and multivariable cox regression analyses for disease-free survival and overall survival in ACRG cohort. | | | | | | | | |
| --- | --- | --- | --- | --- | --- | --- | --- | --- |
| **Variables** | **Disease-free survival (I-III stage, n=257)** | | | | **Overall survival (I-IV stage, n=300)** | | | |
|  | Univariate analysis | | Multivariable analysis | | Univariate analysis | | Multivariable analysis | |
|  | **HR (95%CI)** | **P** | **HR (95%CI)** | **P** | **HR (95%CI)** | **P** | **HR (95%CI)** | **P** |
| **Age** | 1.01 (0.99-1.02) | 0.578 | – | – | 1.01 (0.99-1.03) | 0.181 | – | – |
| **Sex** (female vs. male) | 1.05 (0.69-1.58) | 0.827 | – | – | 0.91 (0.65-1.27) | 0.559 | – | – |
| **Lauren type** (intestinal vs diffuse vs. mixed) | 1.47 (1.03-2.08) | 0.033 | 1.17 (0.79-1.74) | 0.431 | 1.59 (1.22-2.10) | 0.001 | 1.34 (0.98-1.84) | 0.065 |
| **Location** (cardia vs. body vs. antrum vs. whole) | 0.88 (0.67-1.16) | 0.368 | – | – | 0.89 (0.71-1.13) | 0.347 | – | – |
| **TNM stage** (I vs. II vs. III vs. IV) | 2.97 (2.01-4.39) | <0.001 | 3.00 (2.01-4.48) | <0.001 | 2.49 (1.97-3.15) | <0.001 | 2.41 (1.87-3.09) | <0.001 |
| **Chemotherapy** (no vs. yes) | 0.49 (0.33-0.72) | <0.001 | 0.58 (0.46-0.74) | <0.001 | 0.39 (0.28-0.56) | <0.001 | 0.43 (0.30-0.61) | <0.001 |
| **TMEclassifer** |  |  |  |  |  |  |  |  |
| C | 1 | Ref. | 1 | Ref. | 1 | Ref. | 1 | Ref. |
| A | 0.56 (0.31-1.03) | 0.060 | 0.71 (0.37-1.33) | 0.283 | 0.88 (0.48-1.61) | 0.689 | 0.96 (0.52-1.77) | 0.890 |
| B | 0.29 (0.13-0.67) | 0.003 | 0.36 (0.16-0.83) | 0.017 | 0.78 (0.41-1.49) | 0.465 | 0.49 (0.25-0.95) | 0.034 |
| D | 0.66 (0.37-1.19) | 0.169 | 0.71 (0.39-1.27) | 0.245 | 0.86 (0.47-1.60) | 0.652 | 0.86 (0.47-1.59) | 0.637 |

CI, confidence interval; HR, hazard ratio. Patients for disease-free survival assessment had undergone potential radical surgery.

CI, confidence interval; HR, hazard ratio. Patients for disease-free survival assessment had undergone potential radical surgery.

| Table S4. Univariate and multivariable cox regression analyses for disease-free survival and overall survival in SMC cohort. | | | | | | | | |
| --- | --- | --- | --- | --- | --- | --- | --- | --- |
| **Variables** | **Disease-free survival (I-III stage, n=365)** | | | | **Overall survival (I-IV stage, n=432)** | | | |
|  | Univariate analysis | | Multivariable analysis | | Univariate analysis | | Multivariable analysis | |
|  | **HR (95%CI)** | **P** | **HR (95%CI)** | **P** | **HR (95%CI)** | **P** | **HR (95%CI)** | **P** |
| **Age** | 1.00 (0.98-1.01) | 0.820 | – | – | 1.00 (0.98-1.01) | 0.814 | – | – |
| **Sex** (female vs. male) | 1.26 (0.87-1.83) | 0.221 | – | – | 1.04 (0.75-1.42) | 0.830 | – | – |
| **Lauren type** (intestinal vs diffuse vs. mixed) | 0.98 (0.70-1.35) | 0.879 | – | – | 1.18 (0.89-1.58) | 0.254 | – | – |
| **Location** (cardia vs. body vs. antrum vs. whole) | 0.99 (0.79-1.24) | 0.906 | – | – | 0.88 (0.72-1.07) | 0.190 | – | – |
| **TNM stage** (I vs. II vs. III vs. IV) | 2.21 (1.68-2.89) | <0.001 | 2.07 (1.58-2.73) | <0.001 | 2.08 (175-2.47) | <0.001 | 2.03 (1.71-2.42) | <0.001 |
| **Chemotherapy** (no vs. yes) | – | – | – | – | – | – | – | – |
| **TMEclassifer** |  |  |  |  |  |  |  |  |
| A | 1 | Ref. | 1 | Ref. | 1 | Ref. | 1 | Ref. |
| B | 1.24 (0.68-2.27) | 0.479 | 1.09 (0.59-1.99) | 0.780 | 1.25 (0.74-2.12) | 0.400 | 1.09 (0.64-1.84) | 0.762 |
| C | 2.42 (1.43-4.07) | 0.001 | 1.91 (1.13-3.23) | 0.016 | 2.06 (1.29-3.28) | 0.002 | 1.56 (0.96-2.49) | 0.064 |
| D | 2.15 (1.32-3.49) | 0.002 | 1.87 (1.15-2.73) | 0.012 | 2.23 (1.46-3.14) | <0.001 | 1.94 (1.27-2.96) | 0.002 |

| Table S5. Univariate and multivariable cox regression analyses for disease-free survival and overall survival in YUHS cohort. | | | | | | | | |
| --- | --- | --- | --- | --- | --- | --- | --- | --- |
| **Variables** | **Disease-free survival (I-III stage, n=55)** | | | | **Overall survival (I-IV stage, n=59)** | | | |
|  | Univariate analysis | | Multivariable analysis | | Univariate analysis | | Multivariable analysis | |
|  | **HR (95%CI)** | **P** | **HR (95%CI)** | **P** | **HR (95%CI)** | **P** | **HR (95%CI)** | **P** |
| **Age** | 1.02 (0.98-1.05) | 0.459 | – | – | 1.03 (0.99-1.07) | 0.122 | – | – |
| **Sex** (female vs. male) | 0.64 (0.27-1.50) | 0.303 | – | – | 0.67 (0.29-1.54) | 0.347 | – | – |
| **Lauren type** (intestinal vs diffuse vs. mixed) | 0.84 (0.51-1.39) | 0.506 | – | – | 0.78 (0.47-1.31) | 0.350 | – | – |
| **Location** (cardia vs. body vs. antrum vs. whole) | 1.01 (0.62-1.64) | 0.969 | – | – | 0.99 (0.60-1.64) | 0.98 | – | – |
| **TNM stage** (I vs. II vs. III vs. IV) | 2.52 (1.27-5.01) | <0.001 | 2.48 (1.24-4.96) | 0.01 | 2.13 (1.33-3.40) | 0.002 | 2.14 (1.22-3.76) | 0.008 |
| **Chemotherapy** (no vs. yes) | 0.86 (0.53-1.41) | 0.546 | – | – | 0.94 (0.37-2.63) | 0.892 | – | – |
| **TMEclassifer** |  |  |  |  |  |  |  |  |
| C | 1 | Ref. | 1 | Ref. | 1 | Ref. | 1 | Ref. |
| A | 0.49 (0.18-1.38) | 0.497 | 0.72 (0.26-2.01) | 0.529 | 0.55 (0.21-1.48) | 0.239 | 1.44 (0.45-4.65) | 0.539 |
| B | 0.23 (0.05-1.12) | 0.069 | 0.27 (0.06-1.30) | 0.102 | 0.39 (0.10-1.48) | 0.167 | 0.87 (0.20-3.79) | 0.851 |
| D | 0.38 (0.13-1.10) | 0.074 | 0.47 (0.16-1.36) | 0.164 | 0.25 (0.08-0.77) | 0.016 | 0.52 (0.16-1.78) | 0.300 |

CI, confidence interval; HR, hazard ratio. Patients for disease-free survival assessment had undergone potential radical surgery.

| Table S6. Univariate and multivariable cox regression analyses for disease-free survival and overall survival in KUCM cohort. | | | | | | | | |
| --- | --- | --- | --- | --- | --- | --- | --- | --- |
| **Variables** | **Disease-free survival (I-III stage, n=102)** | | | | **Overall survival (I-IV stage, n=109)** | | | |
|  | Univariate analysis | | Multivariable analysis | | Univariate analysis | | Multivariable analysis | |
|  | **HR (95%CI)** | **P** | **HR (95%CI)** | **P** | **HR (95%CI)** | **P** | **HR (95%CI)** | **P** |
| **Age** | 1.07 (1.03-1.10) | <0.001 | 1.06 (1.03-1.09) | <0.001 | 1.04 (1.01-1.07) | 0.005 | 1.04 (1.01-1.07) | 0.017 |
| **Sex** (female vs. male) | 1.34 (0.75-2.39) | 0.321 | – | – | 1.39 (0.78-2.46) | 0.262 | – | – |
| **Lauren type** (intestinal vs diffuse vs. mixed) | 0.81 (0.57-1.16) | 0.247 | – | – | 0.92 (0.67-1.25) | 0.584 | – | – |
| **Location** (cardia vs. body vs. antrum vs. whole) | 1.07 (0.74-1.56) | 0.715 | – | – | 1.24 (0.85-1.82) | 0.262 | – | – |
| **TNM stage** (I vs. II vs. III vs. IV) | 1.89 (1.36-2.62) | <0.001 | 1.74 (1.25-2.43) | 0.001 | 2.41 (1.78-3.28) | <0.001 | 2.38 (1.74-3.26) | <0.001 |
| **Chemotherapy** (no vs. yes) | 1.38 (0.79-2.39) | 0.248 | – | – | 1.51 (0.87-2.58) | 0.129 | – | – |
| **TMEclassifer** |  |  |  |  |  |  |  |  |
| C | 1 | Ref. | 1 | Ref. | 1 | Ref. | 1 | Ref. |
| A | 0.70 (0.32-1.51) | 0.360 | 0.71 (0.33-1.55) | 0.393 | 0.80 (0.38-1.69) | 0.556 | 0.92(0.43-1.96) | 0.822 |
| B | 0.48 (0.18-1.24) | 0.127 | 0.70 (0.28-1.82) | 0.463 | 0.49 (0.19-1.27) | 0.141 | 0.59 (0.23-1.53) | 0.279 |
| D | 0.85 (0.40-1.79) | 0.667 | 0.71 (0.33-1.52) | 0.382 | 0.85 (0.41-1.79) | 0.851 | 0.73 (0.34-1.56) | 0.418 |

CI, confidence interval; HR, hazard ratio. Patients for disease-free survival assessment had undergone potential radical surgery.

| Table S7. Univariate and multivariable cox regression analyses for disease-free survival and overall survival in KUGH cohort. | | | | | | | | |
| --- | --- | --- | --- | --- | --- | --- | --- | --- |
| **Variables** | **Disease-free survival (I-III stage, n=85)** | | | | **Overall survival (I-IV stage, n=93)** | | | |
|  | Univariate analysis | | Multivariable analysis | | Univariate analysis | | Multivariable analysis | |
|  | **HR (95%CI)** | **P** | **HR (95%CI)** | **P** | **HR (95%CI)** | **P** | **HR (95%CI)** | **P** |
| **Age** | 1.01 (0.98-1.05) | 0.442 | – | – | 1.01 (0.98-1.04) | 0.524 | – | – |
| **Sex** (female vs. male) | 0.51 (0.23-1.14) | 0.101 | – | – | 0.60 (0.28-1.26) | 0.174 | – | – |
| **Lauren type** (intestinal vs diffuse vs. mixed) | 1.21 (0.60-2.43) | 0.590 | – | – | 1.44 (0.91-2.29) | 0.122 | – | – |
| **Location** (cardia vs. body vs. antrum vs. whole) | 0.91 (0.55-1.52) | 0.721 | – | – | 1.05 (0.63-1.74) | 0.855 | – | – |
| **TNM stage** (I vs. II vs. III vs. IV) | 2.36 (1.13-4.95) | 0.023 | 2.17 (1.03-4.59) | 0.042 | 3.33 (1.63-6.78) | 0.001 | 2.69 (1.41-5.11) | 0.003 |
| **Chemotherapy** (no vs. yes) | 0.51 (0.23-1.13) | 0.099 | – | – | 0.35 (0.18-0.70) | 0.003 | 0.34 (0.16-0.70) | 0.004 |
| **TMEclassifer** |  |  |  |  |  |  |  |  |
| C | 1 | Ref. | 1 | Ref. | 1 | Ref. | 1 | Ref. |
| A | 0.26 (0.09-0.72) | 0.009 | 0.32 (0.11-0.89) | 0.028 | 0.29 (0.10-0.79) | 0.016 | 0.47 (0.17-1.34) | 0.158 |
| B | 0.38 (0.11-1.27) | 0.116 | 0.42 (0.13-1.40) | 0.158 | 0.39 (0.12-1.30) | 0.126 | 0.68 (0.20-2.33) | 0.539 |
| D | 0.49 (0.19-1.25) | 0.136 | 0.57 (0.23-1.46) | 0.241 | 0.72 (0.30-1.73) | 0.465 | 1.02 (0.41-2.53) | 0.959 |

CI, confidence interval; HR, hazard ratio. Patients for disease-free survival assessment had undergone potential radical surgery.

CI, confidence interval; HR, hazard ratio. Patients for disease-free survival assessment had undergone potential radical surgery.

| Table S8. Univariate and multivariable cox regression analyses for disease-free survival and overall survival in TCGA cohort. | | | | | | | | |
| --- | --- | --- | --- | --- | --- | --- | --- | --- |
| **Variables** | **Disease-free survival (I-III stage, n=329)** | | | | **Overall survival (I-IV stage, n=472)** | | | |
|  | Univariate analysis | | Multivariable analysis | | Univariate analysis | | Multivariable analysis | |
|  | **HR (95%CI)** | **P** | **HR (95%CI)** | **P** | **HR (95%CI)** | **P** | **HR (95%CI)** | **P** |
| **Age** | 0.99 (0.97-1.01) | 0.249 | – | – | 1.02 (1.01-1.04) | 0.012 | 1.03 (1.01-1.05) | <0.001 |
| **Sex** (female vs. male) | 1.77 (1.15-2.71) | 0.009 | 1.80 (1.17-2.78) | 0.008 | 1.28 (0.89-1.82) | 0.178 | – | – |
| **Lauren type** (intestinal vs diffuse vs. mixed) | 1.03 (0.90-1.19) | 0.597 | – | – | 1.06 (0.93-1.20) | 0.387 | – | – |
| **Location** (cardia vs. body vs. antrum vs. whole) | 0.95 (0.79-1.14) | 0.562 | – | – | 0.98 (0.84-1.15) | 0.816 | – | – |
| **TNM stage** (I vs. II vs. III vs. IV) | 1.58 (1.19-2.09) | 0.001 | 1.58 (119-2.10) | 0.002 | 1.57 (1.26-1.96) | <0.001 | 1.71 (1.35-2.16) | <0.001 |
| **Chemotherapy** (no vs. yes) | – | – | – | – | – | – | – | – |
| **TMEclassifer** |  |  |  |  |  |  |  |  |
| A | 1 | Ref. | 1 | Ref. | 1 | Ref. | 1 | Ref. |
| B | 0.69 (0.35-1.35) | 0.279 | 0.59 (0.30-1.17) | 0.131 | 0.86 (0.49-1.53) | 0.615 | 0.69 (0.38-1.25) | 0.225 |
| C | 1.38 (0.80-2.38) | 0.249 | 1.11 (0.64-1.94) | 0.710 | 1.31 (0.78-2.19) | 0.307 | 1.26 (0.75-2.11) | 0.379 |
| D | 1.16 (0.75-1.79) | 0.498 | 1.08 (0.70-1.67) | 0.729 | 1.48 (1.01-2.16) | 0.047 | 1.43 (0.98-2.10) | 0.067 |

CI, confidence interval; HR, hazard ratio

| Table S9. Univariate and multivariable cox regression analyses for overall survival in SGP cohort. | | | | |
| --- | --- | --- | --- | --- |
| **Variables** | Univariate analysis | | Multivariable analysis | |
|  | **HR (95%CI)** | **P** | **HR (95%CI)** | **P** |
| **Age** | 0.997 (0.983-1.012) | 0.704 | – | – |
| **Sex** (female vs. male) | 1.127 (0.775-1.639) | 0.531 | – | – |
| **Lauren type** (intestinal vs diffuse vs. mixed) | 1.161 (0.901-1.497) | 0.249 | – | – |
| **TNM stage** (I vs. II vs. III vs. IV) | 2.444 (1.977-3.021) | <0.001 | 2.396 (1.939-2.961) | <0.001 |
| **TMEclassifer** |  |  |  |  |
| C | 1 | Reference | 1 | Reference |
| A | 0.801 (0.474-1.353) | 0.407 | 0.877 (0.516-1.488) | 0.626 |
| B | 0.456 (0.222-0.937) | 0.033 | 0.545 (0.264-1.125) | 0.100 |
| D | 1.013 (0.619-1.656) | 0.959 | 0.916 (0.559-1.501) | 0.727 |

| Table S10. Univariate and multivariable cox regression analyses for overall survival in MDACC cohort. | | | | |
| --- | --- | --- | --- | --- |
| **Variables** | Univariate analysis | | Multivariable analysis | |
|  | **HR (95%CI)** | **P** | **HR (95%CI)** | **P** |
| **Age** | 0.979 (0.954-1.005) | 0.113 | – | – |
| **Sex** (female vs. male) | 0.859 (0.409-1.802) | 0.688 | – | – |
| **TNM stage** (I vs. II vs. III vs. IV) | 1.955 (1.170-3.269) | 0.011 | 1.910 (1.091-3.343) | 0.024 |
| **TMEclassifer** |  |  |  |  |
| C | 1 | Reference | 1 | Reference |
| A | 0.169 (0.053-0.541) | 0.003 | 0.179 (0.055-0.583) | 0.004 |
| B | 0.710 (0.198-2.550) | 0.600 | 0.485 (0.128-1.183) | 0.286 |
| D | 0.233 (0.071-0.763) | 0.016 | 0.199 (0.060-0.663) | 0.009 |

CI, confidence interval; HR, hazard ratio

| Table S11. Univariate and multivariable cox regression analyses for overall survival in TYB cohort. | | | | |
| --- | --- | --- | --- | --- |
| **Variables** | Univariate analysis | | Multivariable analysis | |
|  | **HR (95%CI)** | **P** | **HR (95%CI)** | **P** |
| **Age** | 1.018 (0.995-1.041) | 0.123 | – | – |
| **Sex** (female vs. male) | 0.891 (0.549-1.448) | 0.642 | – | – |
| **TNM stage** (I vs. II vs. III vs. IV) | 1.155 (0.794-1.680) | 0.451 | 1.285 (0.879 -1.877) | 0.195 |
| **TMEclassifer** |  |  |  |  |
| C | 1 | Reference | 1 | Reference |
| A | 0.608 (0.364-1.018) | 0.059 | 0.575 (0.341-0.968) | 0.037 |
| B | 0.346 (0.149-0.800) | 0.013 | 0.314 (0.134-0.735) | 0.008 |
| D | 0.571 (0.336-0.971) | 0.038 | 0.544 (0.319-0.928) | 0.025 |

CI, confidence interval; HR, hazard ratio

| Table S12. Univariate and multivariable cox regression analyses for overall survival in CGH cohort. | | | | |
| --- | --- | --- | --- | --- |
| **Variables** | Univariate analysis | | Multivariable analysis | |
|  | **HR (95%CI)** | **P** | **HR (95%CI)** | **P** |
| **Age** | 1.005 (0.989-1.022) | 0.529 | – | – |
| **Sex** (female vs. male) | 0.755 (0.477-1.195) | 0.231 | – | – |
| **Lauren type** (intestinal vs diffuse vs. mixed) | 0.999 (0.753-1.326) | 0.996 | – | – |
| **TNM stage** (I vs. II vs. III vs. IV) | 3.376 (2.413-4.724) | <0.001 | 3.483 (2.451-4.951) | <0.001 |
| **TMEclassifer** |  |  |  |  |
| C | 1 | Reference | 1 | Reference |
| A | 1.068 (0.660-1.728) | 0.789 | 1.169 (0.720-1.898) | 0.527 |
| B | 0.839 (0.428-1.644) | 0.608 | 1.377 (0.683-2.773) | 0.371 |
| D | 0.723 (0.415-1.259) | 0.251 | 0.837 (0.478-1.464) | 0.532 |

CI, confidence interval; HR, hazard ratio

| Table S13. Univariate and multivariable cox regression analyses for overall survival in KOREA cohort. | | | | |
| --- | --- | --- | --- | --- |
| **Variables** | Univariate analysis | | Multivariable analysis | |
|  | **HR (95%CI)** | **P** | **HR (95%CI)** | **P** |
| **Age** | 1.019 (1.006-1.031) | 0.003 | – | – |
| **Sex** (female vs. male) | 1.254 (0.926-1.697) | 0.143 | – | – |
| **T stage** (T1 vs T2 vs. T3 vs. T4) | 1.699 (1.351-2.137) | <0.001 | 1.489 (1.173-1.889) | 0.001 |
| **N stage** (N0 vs. N1 vs. N2 vs. N3) | 1.664 (1.418-1.952) | <0.001 | 1.549 (1.312-1.830) | <0.001 |
| **TMEclassifer** |  |  |  |  |
| C | 1 | Reference | 1 | Reference |
| A | 0.797 (0.523-1.215) | 0.292 | 0.743 (0.486-1.134) | 0.169 |
| B | 0.325 (0.169-0.623) | 0.001 | 0.322 (0.168-0.618) | 0.001 |
| D | 0.906 (0.599-1.372) | 0.643 | 0.758 (0.499-1.150) | 0.193 |

CI, confidence interval; HR, hazard ratio

| Table S14. Univariate cox regression analyses for disease-free survival and overall survival in gastric cancer meta-cohort. | | | | |
| --- | --- | --- | --- | --- |
| **Variables** | **Disease-free survival** | | **Overall survival** | |
|  | **HR (95%CI)** | **P** | **HR (95%CI)** | **P** |
| **Age** | 1.014 (1.006-1.022) | 0.001 | 1.015 (1.010-1.021) | <0.001 |
| **Sex** (female vs. male) | 1.211 (0.990-1.482) | 0.062 | 1.113 (0.981-1.263) | 0.097 |
| **Lauren type** |  |  |  |  |
| Intestinal | 1 | Reference | 1 | Reference |
| Diffuse | 1.048 (0.861-1.274) | 0.642 | 1.173 (0.985-1.396) | 0.073 |
| Mixed | 0.953 (0.554-1.638) | 0.861 | 0.926 (0.566-1.514) | 0.759 |
| **Location** |  |  |  |  |
| Cardia | 1 | Reference | 1 | Reference |
| Body | 0.905 (0.688-1.191) | 0.476 | 0.801 (0.628-1.023) | 0.076 |
| Antrum | 0.851 (0.657-1.104) | 0.225 | 0.733 (0.581-0.924) | 0.009 |
| Whole | 0.806 (0.372-1.746) | 0.584 | 1.025 (0.586-1.793) | 0.932 |
| **TNM stage** |  |  |  |  |
| I | 1 | Reference | 1 | Reference |
| II | 2.264 (1.608-3.189) | <0.001 | 2.134 (1.591-2.861) | <0.001 |
| III | 4.835(3.397-6.881) | <0.001 | 4.664 (3.508-6.201) | <0.001 |
| IV | – | – | 9.250 (6.825-12.536) | <0.001 |
| **Chemotherapy** (no vs. yes) | 0.586 (0.465-0.740) | <0.001 | 0.507 (0.415-0.620) | <0.001 |
| **TMEclassifer** |  |  |  |  |
| A | 1 | Reference | 1 | Reference |
| B | 0.743 (0.539-1.025) | 0.070 | 0.740 (0.608-0.901) | 0.003 |
| C | 1.680 (1.290-2.187) | <0.001 | 1.355 (1.145-1.604) | <0.001 |
| D | 1.347 (1.074-1.690) | 0.010 | 1.186 (1.030-1.364) | 0.018 |

CI, confidence interval; HR, hazard ratio. Patients for disease-free survival assessment had undergone potential radical surgery.

| Table S15. Univariable and multivariable logistic regression analyses for recurrence of patients with gastric cancer in meta-cohort A. | | | | |
| --- | --- | --- | --- | --- |
| **Variables** | **Univariate analysis** | | **Multivariable analysis** | |
|  | **OR (95%CI)** | **P** | **OR (95%CI)** | **P** |
| **Age** | 0.999 (0.989-1.008) | 0.767 | – | – |
| **Sex** (female vs. male) | 1.247 (0.969-1.604) | 0.086 | – | – |
| **Lauren type** |  |  |  |  |
| Intestinal | 1 | Reference |  |  |
| Diffuse | 1.222 (0.950-1.571) | 0.118 | – | – |
| Mixed | 1.040 (0.524-2.067) | 0.910 | – | – |
| **Location** |  |  |  |  |
| Cardia | 1 | Reference | – | – |
| Body | 1.212 (0.857-1.714) | 0.278 | – | – |
| Antrum | 1.046 (0.756-1.448) | 0.787 | – | – |
| Whole | 1.238 (0.453-3.383) | 0.677 | – | – |
| **TNM stage** |  |  |  |  |
| I | 1 | Reference | 1 | Reference |
| II | 2.679 (1.820-3.945) | <0.001 | 3.285 (2.065-5.227) | <0.001 |
| III | 4.346 (2.886-6.546) | <0.001 | 5.611 (3.379-9.318) | <0.001 |
| **Chemotherapy** (no vs. yes) | 0.673 (0.493-0.918) | 0.012 | 0.581 (0.408-0.828) | 0.003 |
| **TMEclassifer** |  |  |  |  |
| A | 1 | Reference | 1 | Reference |
| B | 0.739 (0.506-1.079) | 0.118 | 0.727 (0.464-1.140) | 0.165 |
| C | 2.303 (1.608-3.298) | <0.001 | 2.707 (1.745-4.200) | <0.001 |
| D | 1.492 (1.121-1.984) | 0.006 | 1.490 (1.046-2.121) | 0.027 |

CI, confidence interval; HR, hazard ratio

| Table S16. The statistical comparisons in subgroups of TME-classifier about stage, recurrence, histology, mutation burden and TMB | | | | | | | |
| --- | --- | --- | --- | --- | --- | --- | --- |
| **Variables** | TMEclassifier-A+ B**:** TMEclassifier C+D | TMEclassifier-A**:** B | TMEclassifier-A**:** C | TMEclassifier-A**:** D | TMEclassifier-B**:** C | TMEclassifier-B**:** D | TMEclassifier-C**:** D |
|  | P value | P value | P value | P value | P value | P value | P value |
| Tumor staging | <0.001 | 0.298 | 0.003 | 0.001 | 0.130 | 0.038 | 0.197 |
| Recurrence status | <0.001 | 0.117 | <0.001 | 0.006 | <0.001 | <0.001 | 0.015 |
| Tumor location | 0.726 | 0.171 | 0.532 | 0.060 | 0.325 | 0.298 | 0.013 |
| Histology | <0.001 | 0.001 | <0.001 | <0.001 | 0.505 | <0.001 | 0.048 |
| Mutation burden | <0.001 | 0.772 | 0.014 | <0.001 | 0.039 | 0.002 | 0.319 |
| TMB | <0.001 | 0.713 | 0.012 | <0.001 | 0.024 | 0.002 | 0.440 |


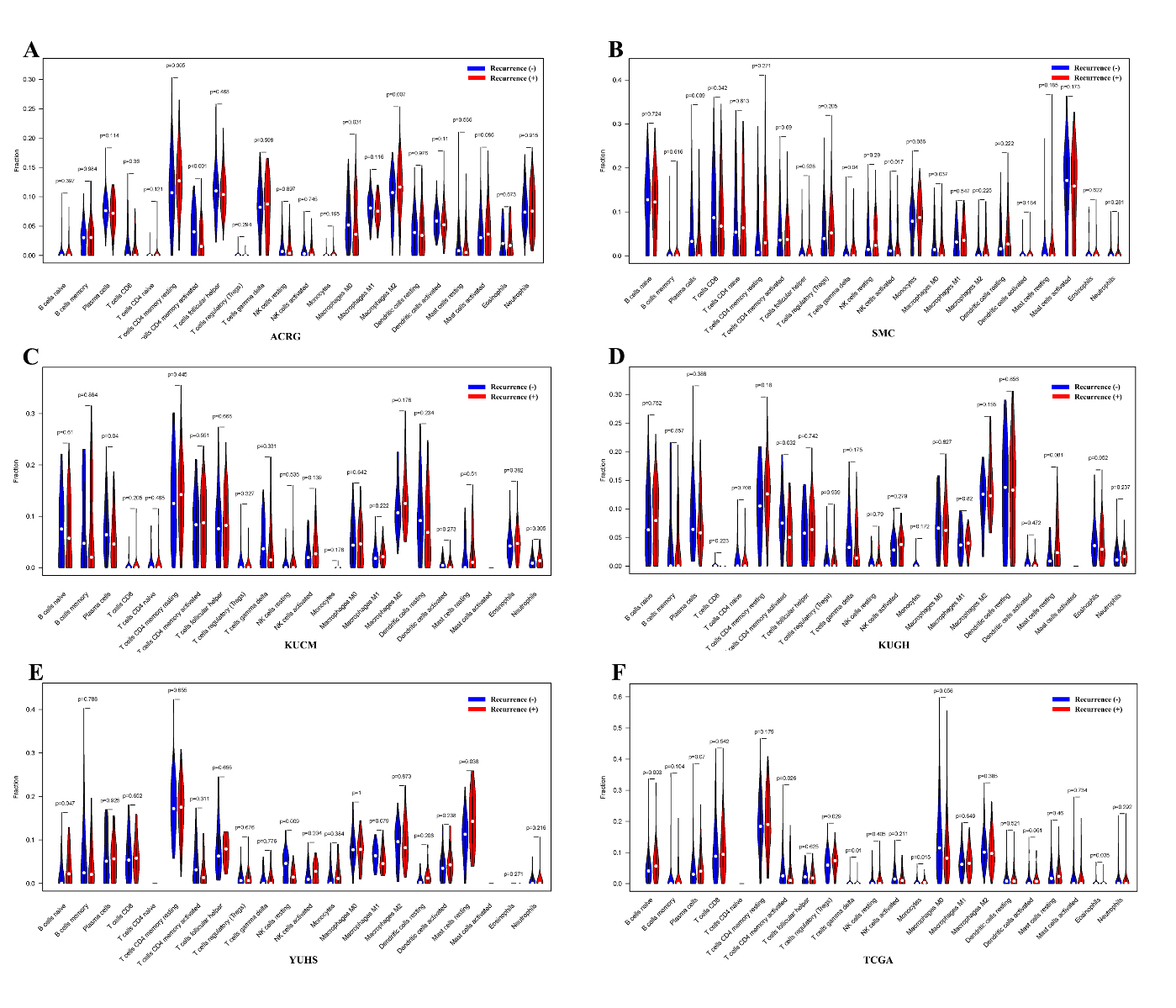


Figure S1. 22 tumor-associated infiltrating immune cells in ACRG (A), SMC (B), KUCM (C), KUGH (D), YUHS (E) and TCGA -STAD (F) by CYBERSORT algorithm.


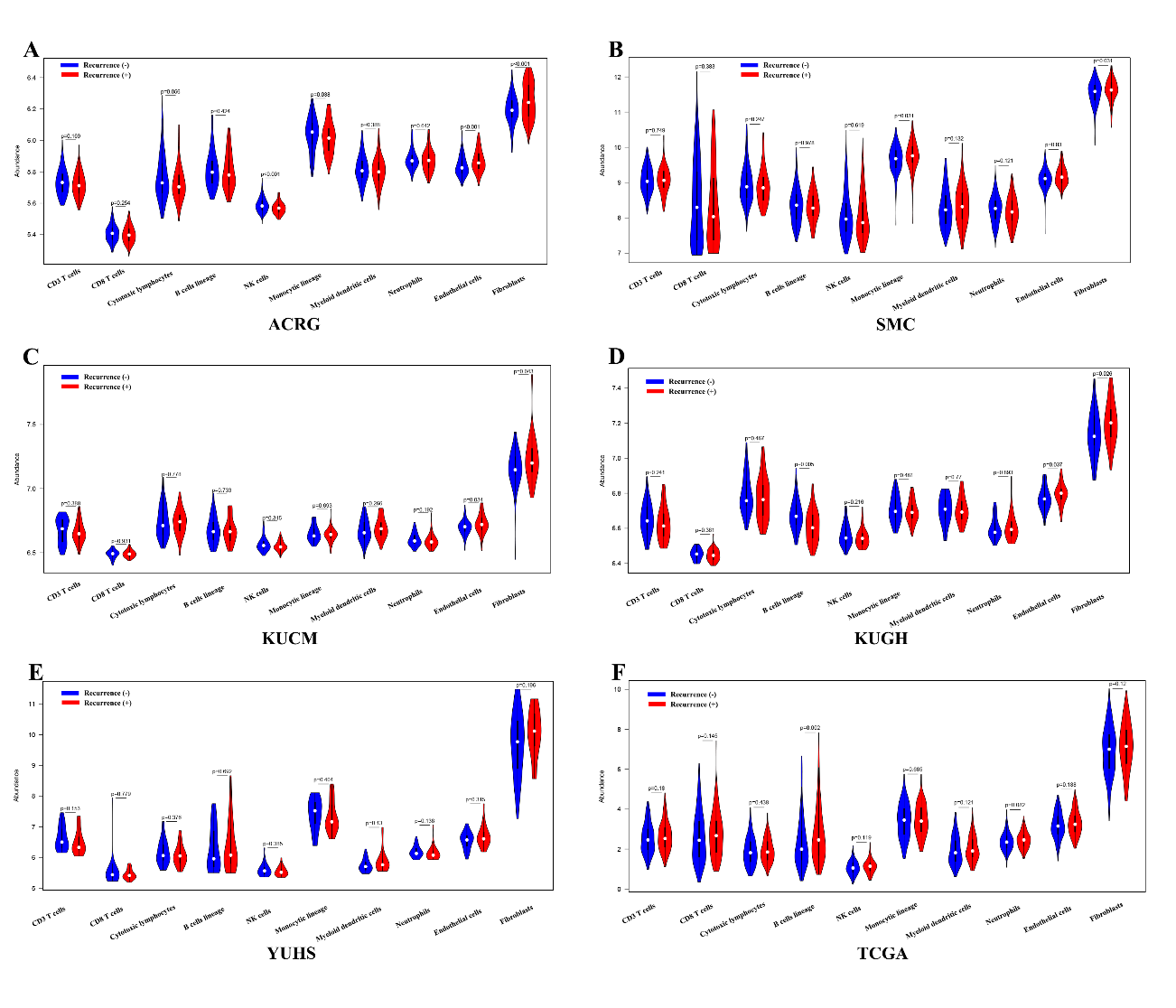


Figure S2. 10 tumor-associated infiltrating immune-stromal cells in ACRG (A), SMC (B), KUCM (C), KUGH (D), YUHS (E) and TCGA -STAD (F) by MCPcounter algorithm.


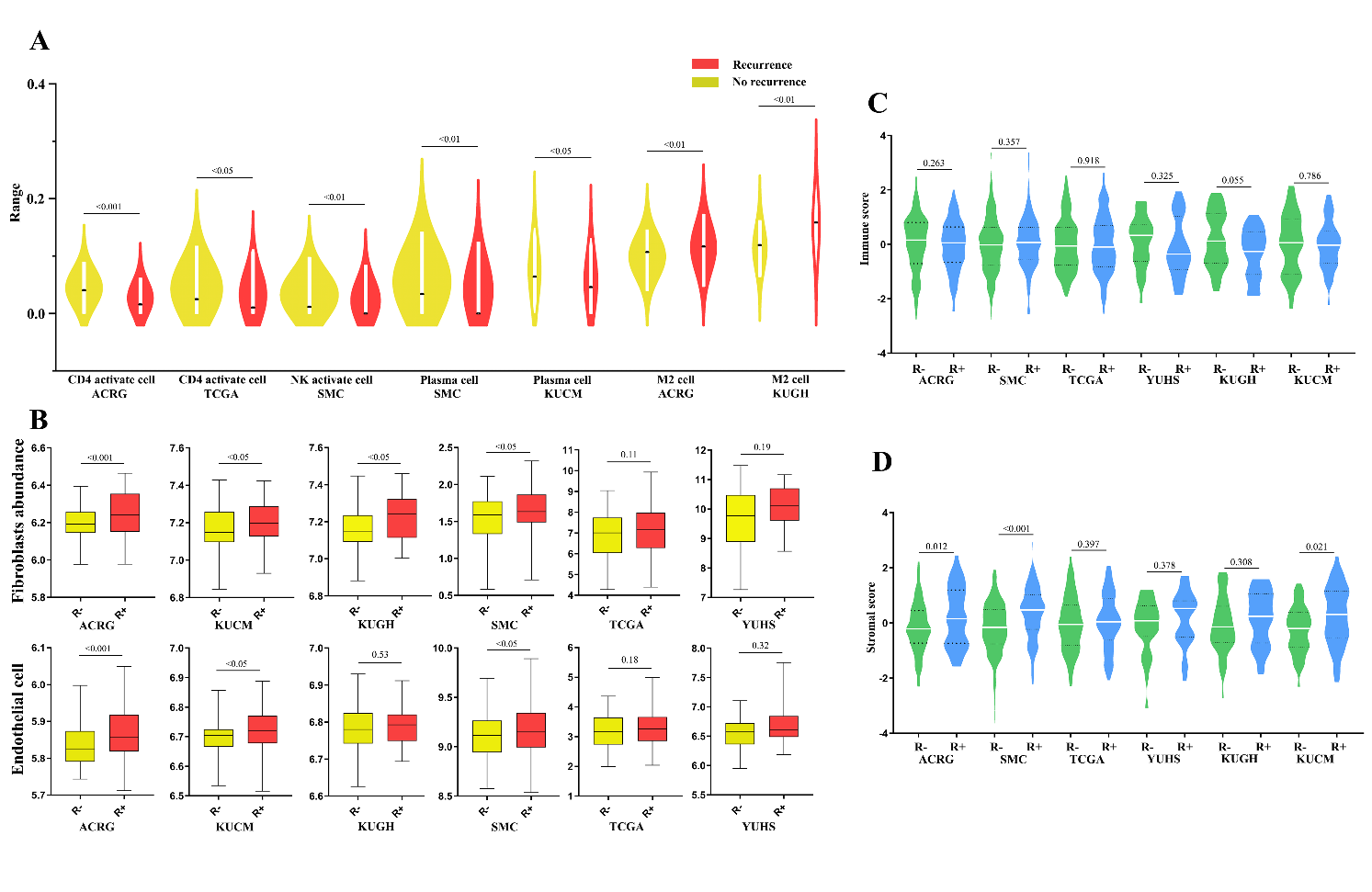


Figure S3. Tumor-associated infiltrating immune cells with significance in ACRG, SMC, KUCM, KUGH, and TCGA-STAD (A). Fibroblasts and endothelial cells (B) in ACRG, SMC, KUCM, KUGH, YUHS, and TCGA-STAD. Immune score (C) and stromal score (D) in ACRG, SMC, KUCM, KUGH, YUHS, and TCGA-STAD.


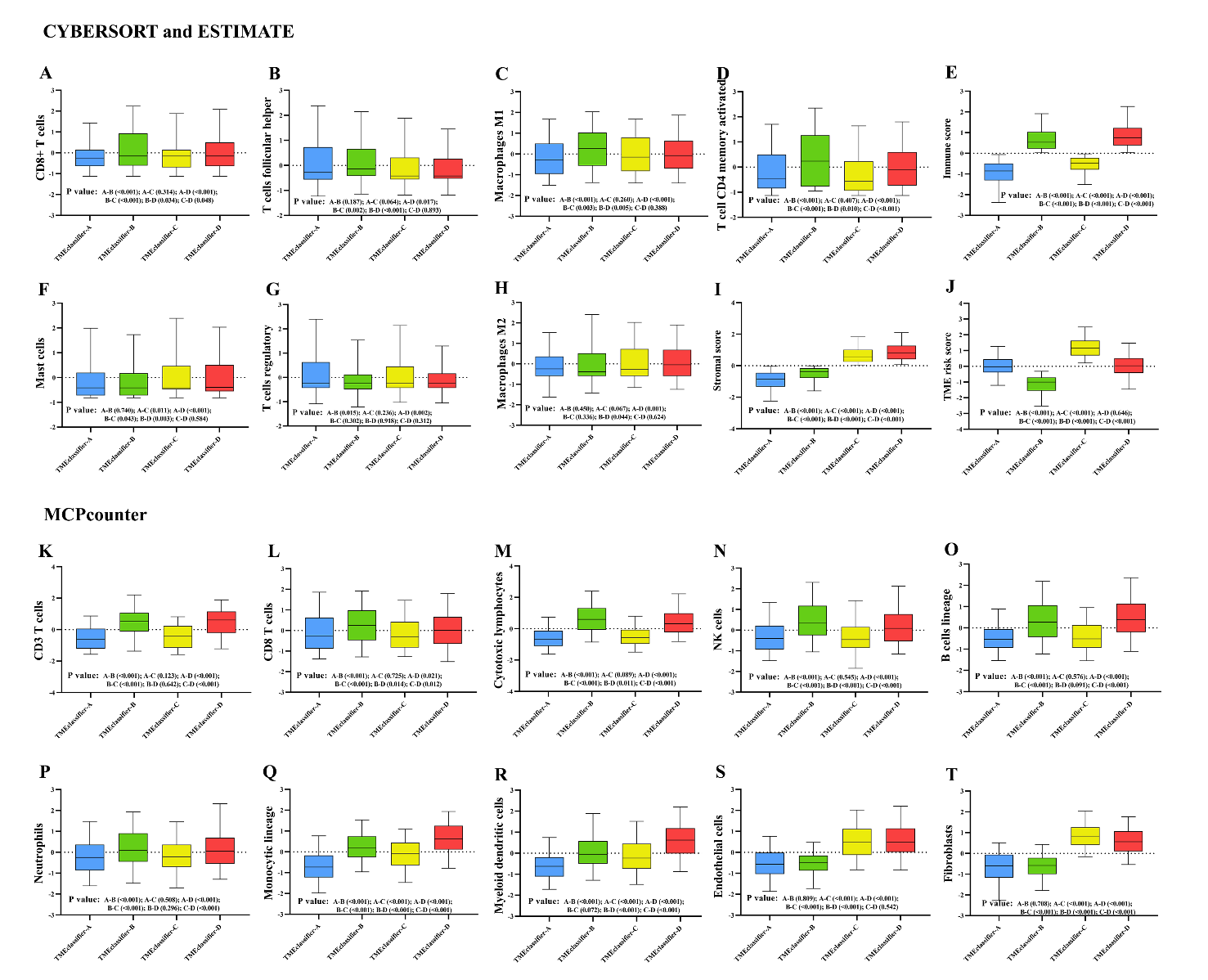
Figure S4. The proportion of immune-stromal cells and immune-stromal score based on CYBERSORT, MCPcounter and ESTIMATE algorithms stratified by the TME-classifier.


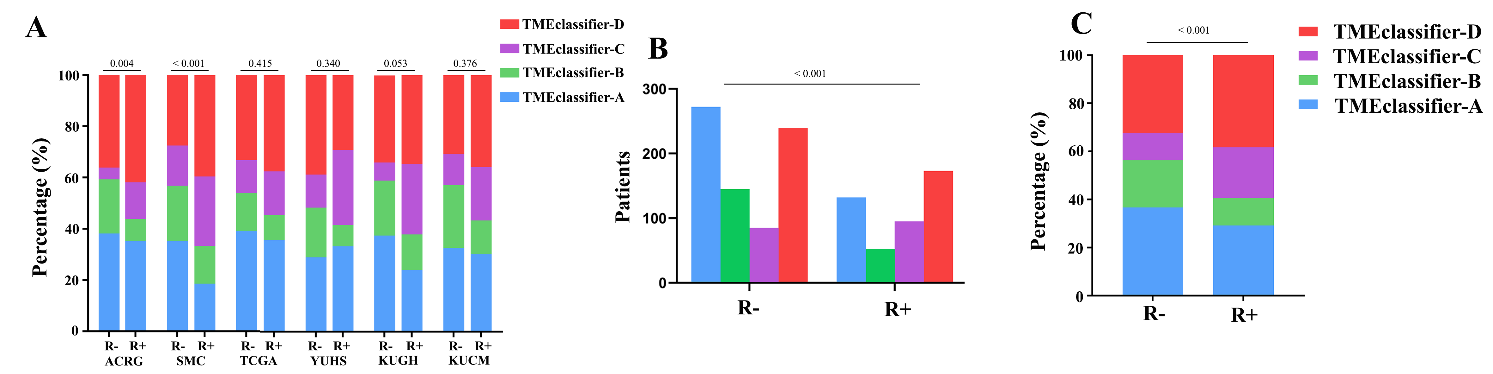


Figure S5. The proportion (A) of 4 subtypes of TME-classifier in ACRG, SMC, KUCM, KUGH, YUHS, and TCGA-STAD based on tumor recurrence status. 4 subtypes of TME-classifier stratified by recurrence status in the meta-cohort A (B and C).


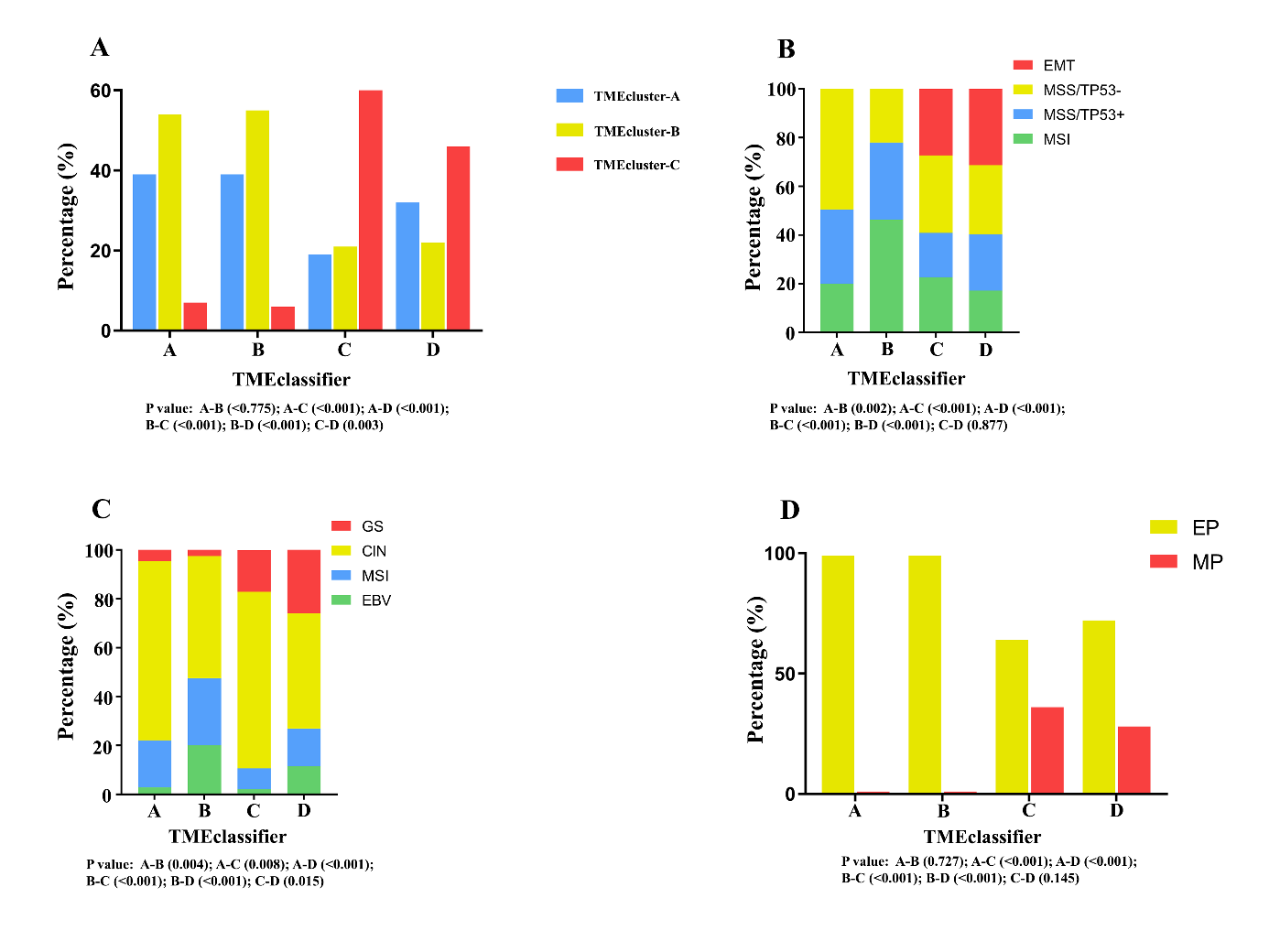


Figure S6. Histograms of TME-classifier in groups with different TME clusters (A), ACRG subtypes (B), TCGA subtypes (C) and EM subtypes (D).


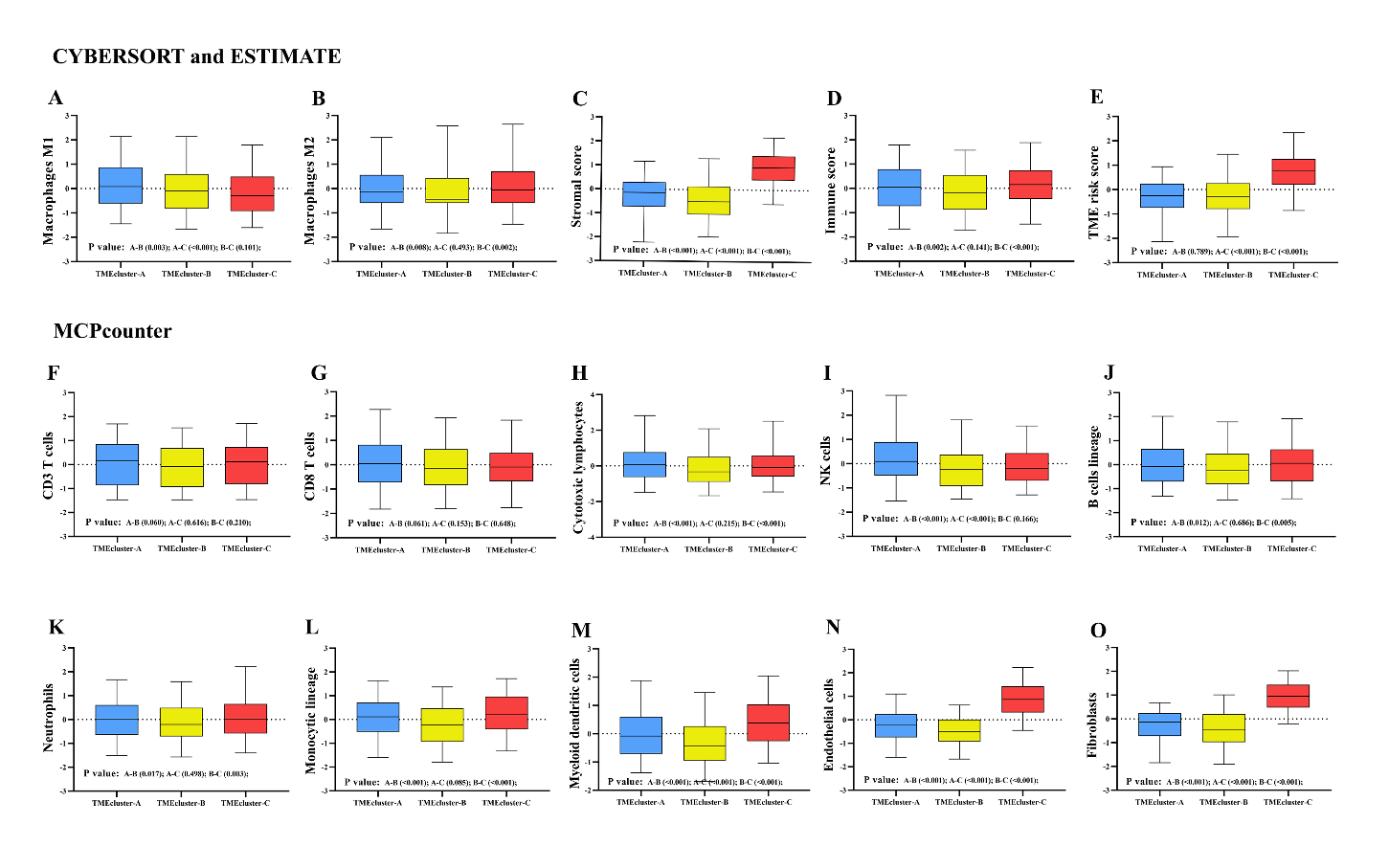
Figure S7. The proportion of immune-stromal cells and immune-stromal score based on CYBERSORT, MCPcounter and ESTIMATE algorithms stratified by the TME-cluster.


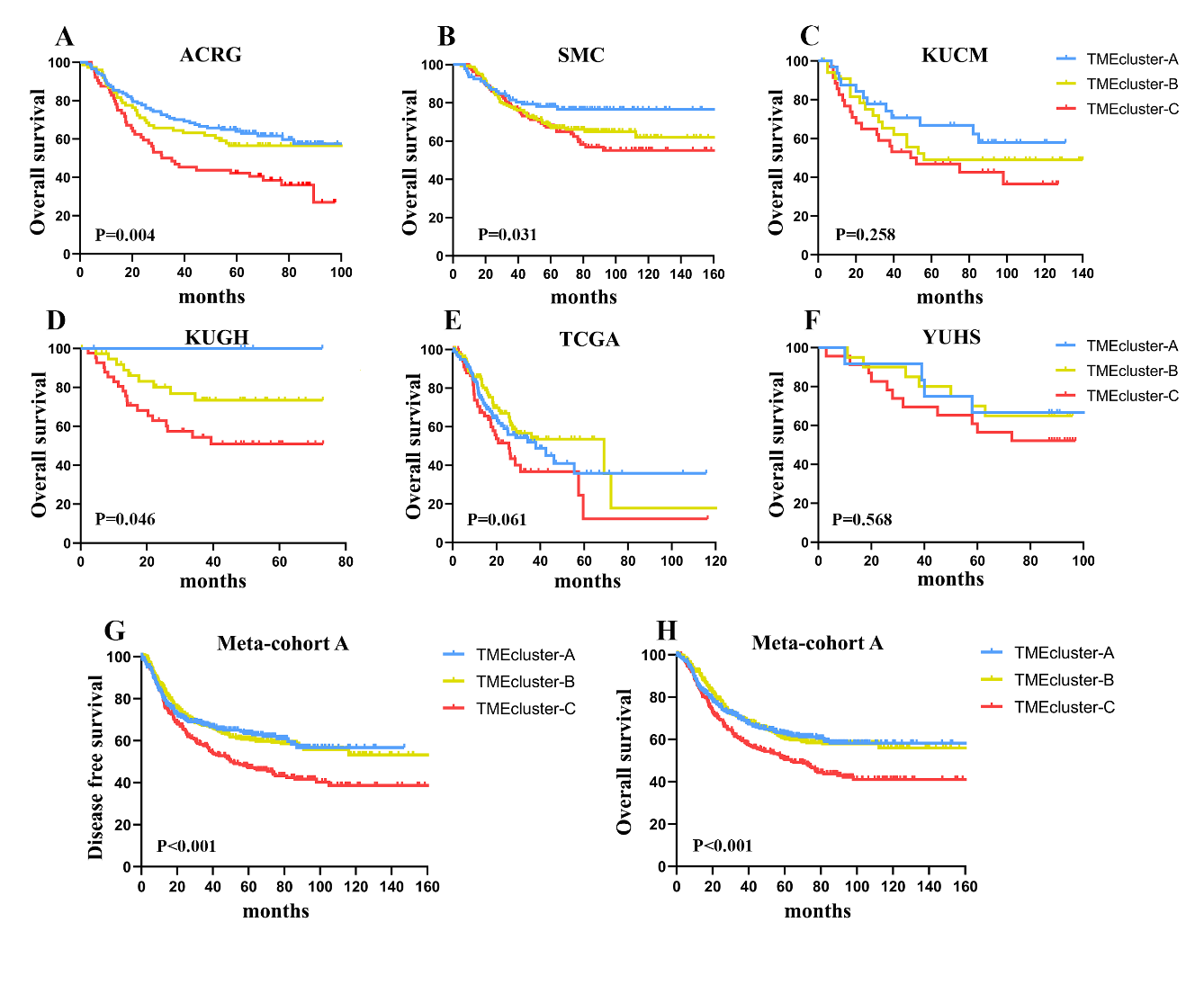


Figure S8. Kaplan–Meier curves for overall survival of patients with stage I–III gastric cancer based on TME-cluster in the ACRH, SMC, KUCM, KUGH, TCGA, YUHS, meta-cohort A. Kaplan–Meier curves for disease-free survival of patients with stage I–III gastric cancer based on TME-cluster in the meta-cohort A.


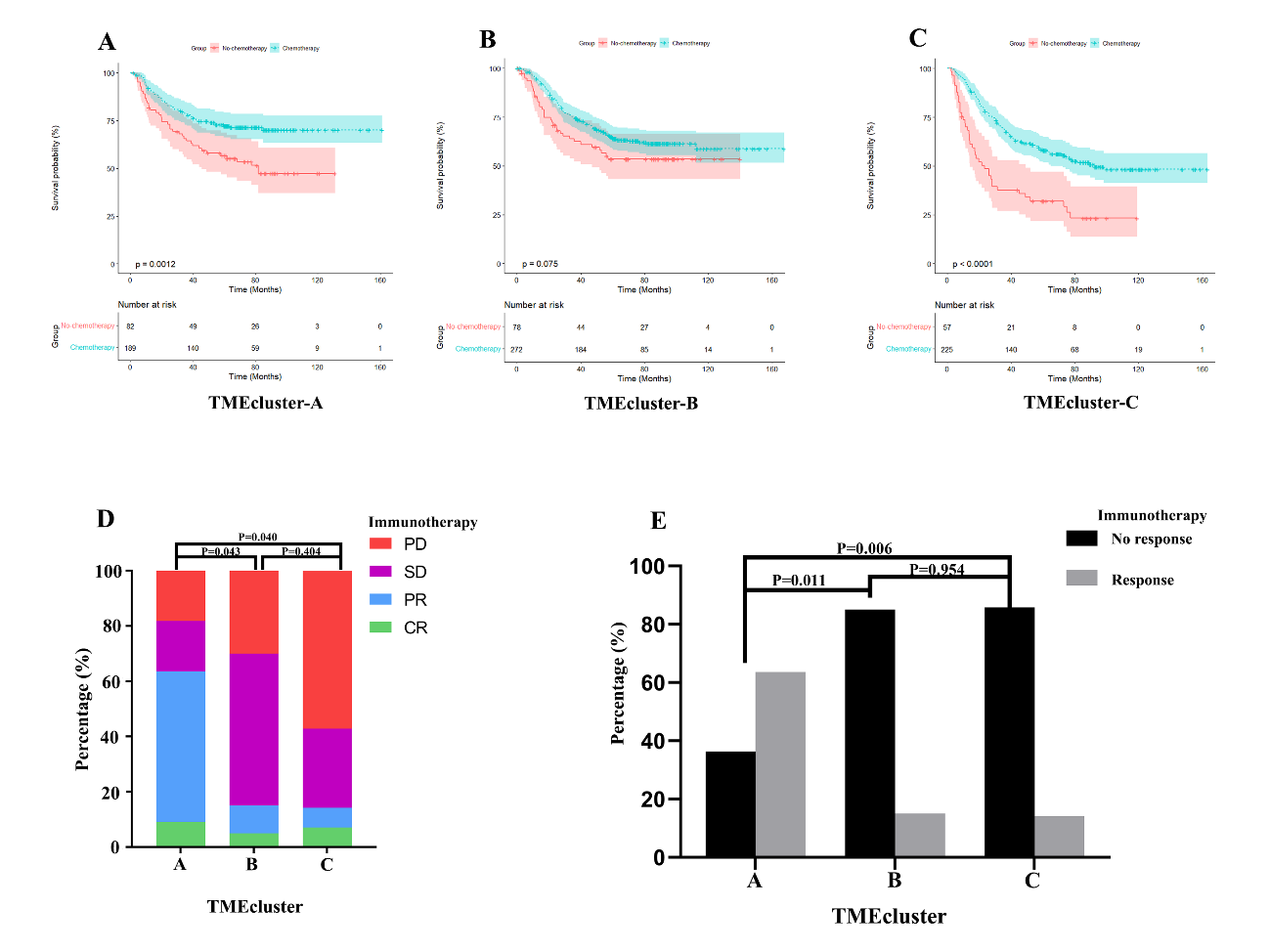


Figure S9. Predictive relevance of the TME-cluster for the benefit of chemotherapy in stage I–III gastric cancer. Patients of TME-cluster A and C derived a significant survival benefit from adjuvant chemotherapy (A and C). However, patients of TME-cluster B did not benefit from adjuvant chemotherapy (B). Patients in TME-classifier A group respond best to pembrolizumab treatment (PD-1 inhibitor), followed by TMEclassifier-B and TME-classifier group (D and E).

**III Supplementary Reference**

**References:**

1. Newman AM, Liu CL, Green MR, Gentles AJ, Feng W, Xu Y, et al. Robust enumeration of cell subsets from tissue expression profiles. Nat Methods. 2015;12(5):453-7. Epub 2015/03/31. doi: 10.1038/nmeth.3337. PubMed PMID: 25822800; PubMed Central PMCID: PMCPMC4739640.

2. Becht E, Giraldo NA, Lacroix L, Buttard B, Elarouci N, Petitprez F, et al. Estimating the population abundance of tissue-infiltrating immune and stromal cell populations using gene expression. Genome Biol. 2016;17(1):218. Epub 2016/10/22. doi: 10.1186/s13059-016-1070-5. PubMed PMID: 27765066; PubMed Central PMCID: PMCPMC5073889.

3. Yoshihara K, Shahmoradgoli M, Martinez E, Vegesna R, Kim H, Torres-Garcia W, et al. Inferring tumour purity and stromal and immune cell admixture from expression data. Nat Commun. 2013;4:2612. Epub 2013/10/12. doi: 10.1038/ncomms3612. PubMed PMID: 24113773; PubMed Central PMCID: PMCPMC3826632.

4. Yu G, Wang LG, Han Y, He QY. clusterProfiler: an R package for comparing biological themes among gene clusters. OMICS. 2012;16(5):284-7. Epub 2012/03/30. doi: 10.1089/omi.2011.0118. PubMed PMID: 22455463; PubMed Central PMCID: PMCPMC3339379.
